# Beyond Accuracy: A Controlled Comparative Glaucoma Screening Benchmark Across Deep Learning and Hybrid Models Under Within- and Cross-Dataset Conditions

**DOI:** 10.3390/jcm15166418

**Published:** 2026-08-19

**Authors:** Haifa F. Alhasson, Shuaa S. Alharbi, Muhammed S. Alluwimi

**Affiliations:** 1 Department of Information Technology, College of Computer, Qassim University, Buraydah 52571, Al-Qassim, Saudi Arabia; shuaa.s.alharbi@qu.edu.sa; 2 Department of Optometry, College of Applied Medical Sciences, Qassim University, Buraydah 52571, Al-Qassim, Saudi Arabia; m.alluwimi@qu.edu.sa

**Keywords:** glaucoma screening, retinal fundus imaging, deep learning, semi-supervised learning, transfer learning, cross-dataset validation, calibration, benchmark analysis

## Abstract

**Background/Objectives**: Artificial intelligence (AI) models for glaucoma screening using colour fundus photography have shown strong internal performance; however, their external validity and calibration reliability remain uncertain. This study developed a controlled four-dataset benchmark to evaluate six glaucoma-screening models across RIM-ONE, DRISHTI-GS, the Hillel Yaffe Glaucoma Dataset, and ORIGA. **Methods**: The evaluated models included a hybrid deep-handcrafted random forest (RF), transfer-learning and semi-supervised VGG16 models, and a compact convolutional neural network (CNN). Performance was assessed in within-dataset and cross-dataset settings using discrimination metrics, including accuracy, area under the receiver operating characteristic curve (AUC), balanced accuracy, and Matthews correlation coefficient (MCC), as well as calibration metrics, including Brier score and expected calibration error (ECE). Threshold stability, preprocessing ablation, and repeated-seed analyses were also performed. **Results**: Within-dataset evaluation showed strong discrimination, with the complete hybrid CNN + histogram of oriented gradients (HOG) + local binary patterns (LBP) + minimum redundancy maximum relevance (mRMR) + RF pipeline achieving a mean accuracy of 0.8697 and an AUC of 0.8972. However, cross-dataset performance was poor, with a mean accuracy of 0.5685 and an AUC of 0.5798. The denoising autoencoder-enhanced transfer-learning model showed improved probability calibration in transfer settings. Preprocessing effects were dataset-dependent, with raw images, region of interest (ROI) cropping, and Retinex normalisation producing different external performance patterns. **Conclusions**: High internal accuracy did not translate into reliable cross-domain generalisation. None of the evaluated models were suitable for zero-shot deployment without site-specific validation or recalibration.

## 1. Introduction

Glaucoma is a chronic, progressive optic neuropathy characterised by retinal ganglion cell loss and structural damage to the optic nerve head, and it remains one of the leading causes of irreversible blindness worldwide [1,2,3]. Because the disease often develops insidiously before patients experience functionally significant visual symptoms, timely screening and early referral are essential for reducing preventable visual loss [4]. In this context, colour fundus photography remains clinically attractive as a non-invasive, relatively inexpensive, and scalable imaging modality for population-level screening, particularly where access to more specialised structural testing is limited [5,6]. Structural changes in the optic nerve head and peripapillary region may precede detectable visual field loss, making retinal imaging a valuable substrate for early risk assessment. However, manual interpretation of fundus photographs is laborious, subjective, and sensitive to inter-observer variability, which has motivated growing interest in artificial intelligence (AI) methods for more reproducible glaucoma screening [2,7].

The clinical promise of fundus-based AI must nevertheless be interpreted with caution. Glaucoma diagnosis from disc photographs often relies on morphometric indicators such as the cup-to-disc ratio, yet these measures can be unreliable in eyes with large or small discs, high myopia, tilted discs, variable illumination, or other anatomical and acquisition-related sources of ambiguity [1,4,5]. More broadly, fundus-image analysis is influenced by camera characteristics, field of view, colour balance, illumination non-uniformity, image quality, and grading conventions, all of which can alter the apparent distribution seen by an algorithm. As a result, models that perform strongly on a single internal test set may deteriorate when transferred to data from a different acquisition domain or clinical source [8,9]. This challenge is especially important in glaucoma screening, where deployment decisions depend not only on discrimination performance, but also on calibration quality, threshold portability, and preservation of sensitivity-specificity balance across populations.

A wide range of algorithmic strategies has therefore been explored for automated glaucoma screening. Deep learning models can learn hierarchical and task-specific representations directly from retinal images, while classical machine-learning methods remain appealing when labelled data are limited or when computational efficiency is a priority [10,11]. Semi-supervised approaches are particularly relevant in ophthalmic imaging because expert-labelled data are costly to obtain, whereas unlabelled fundus photographs are comparatively abundant [12,13]. At the same time, compact architectures and hybrid frameworks that combine handcrafted and learned representations remain important for edge deployment, lower-resource screening settings, and scenarios in which interpretability and latency are clinically relevant considerations alongside predictive accuracy [14,15]. Yet despite this methodological diversity, fair comparison across model families remains difficult because published studies frequently differ in data partitioning, preprocessing, augmentation, threshold selection, and evaluation metrics [1,2,3,16].

A persistent limitation of the glaucoma-AI literature is that many reported gains are established under narrow experimental conditions. Single-dataset and single-run evaluations remain common, and relatively few studies systematically examine seed sensitivity, calibration, threshold stability, or cross-dataset transportability under a common protocol [8,9]. Consequently, it is often unclear whether apparent improvements reflect genuine methodological superiority or are instead driven by favourable splits, preprocessing choices, prevalence structure, or source-specific image characteristics. This is a significant practical problem: in screening applications, a modestly lower internal AUC may be more acceptable than a model whose operating characteristics become unstable after transfer to a new clinic, camera, or population.

The present study addresses this gap through a controlled benchmark of six model families evaluated across four public glaucoma datasets—RIM-ONE, DRISHTI-GS, HYGD, and ORIGA—under both within-dataset and cross-dataset conditions. Rather than introducing yet another classification architecture, the study focuses on rigorous comparative evaluation under clinically relevant heterogeneity in image appearance, disease prevalence, and acquisition domain. To our knowledge, few prior studies have compared transfer-learning, semi-supervised, compact, and hybrid approaches within a single harmonised framework that simultaneously incorporates repeated-seed evaluation, preprocessing ablation, validation-based threshold tuning, calibration analysis, balanced sampling, test-time augmentation, and explicit cross-dataset transfer assessment. By placing these methodological families under a common protocol, the benchmark reveals distinctions that are often obscured in conventional single-split reporting, including differences in robustness, probability reliability, and operational stability.

Existing studies usually focus on proposing a new architecture or reporting high internal accuracy. In contrast, this study is novel because it systematically benchmarks multiple model families under the same protocol across four datasets, four preprocessing pipelines, repeated seeds, calibration, threshold stability, computational cost, and twelve zero-shot cross-dataset transfer tasks.

This work makes four principal contributions. First, it establishes a reproducible four-dataset evaluation framework for glaucoma screening from fundus images, enabling fairer comparison across heterogeneous methodological families and providing a stronger basis for future benchmarking studies. Second, it evaluates the effect of multiple preprocessing strategies across both internal and transfer settings, thereby clarifying when region-of-interest extraction or illumination normalisation improves performance and when such interventions may be neutral or detrimental. Third, it extends model assessment beyond discrimination metrics by incorporating calibration error, Brier score, threshold stability, and non-parametric statistical testing, thereby operationalising a deployment-oriented view of model quality rather than an accuracy-only perspective. Fourth, it relates predictive performance to computational cost, inference latency, and training burden, yielding a multi-dimensional comparison that is more aligned with the realities of ophthalmic screening deployment, especially in settings where computational resources and specialist oversight may be limited.

It is important to emphasise that the primary novelty of this study lies not in a new classification architecture, but in the design and joint execution of an evaluation protocol whose individual elements have appeared separately, but not in combination, in prior glaucoma-screening research. Specifically, while cross-dataset evaluation, calibration reporting, and repeated-seed analysis have each been explored in isolation, no previous study has, to our knowledge, combined four heterogeneous public datasets, four preprocessing configurations, five repeated random seeds, validation-based threshold optimisation, calibration and threshold-stability analysis, and non-parametric statistical testing within a single unified benchmark spanning three distinct methodological families. This combination is what allows the present study to reveal differences in model trustworthiness that remain hidden under conventional single-dataset, single-split reporting. Taken together, this benchmark is designed to answer a question of greater practical importance than which model attains the highest internal score on a single dataset: which class of method remains most trustworthy when the evaluation setting becomes more realistic. By emphasising external validity, calibration, and operational behaviour alongside discrimination performance, the study aims to provide a more clinically meaningful evidence base for selecting glaucoma-screening models for multicenter validation and eventual translation.

## 2. Literature Review

The literature on automated glaucoma screening from fundus photographs is extensive and technically diverse, spanning classical machine learning, deep convolutional networks, transfer learning, semi-supervised strategies, hybrid feature fusion, and attention-based architectures. Rather than cataloguing these contributions chronologically, this review organises prior work around four research problems that together motivate the benchmark design of the present study: the characteristics and limitations of public glaucoma datasets; the methodological landscape of fundus-based AI screening; the underexplored challenge of external validation, domain shift, and calibration; and the clinical context defined by the comparative strengths and weaknesses of fundus photography and optical coherence tomography as screening modalities. In response to the rapid expansion of the field, we place particular emphasis on studies published during 2024–2026, including recent work on external validation, domain adaptation and domain generalisation, transformer-based and attention-based models, self-supervised and semi-supervised learning, multimodal fusion, explainable AI, and hybrid deep-handcrafted feature approaches.

### 2.1. Public Glaucoma Datasets: Scope, Heterogeneity, and Limitations

Automated glaucoma screening research has depended heavily on a small number of publicly available fundus image datasets, and the properties of these datasets profoundly shape both the methods developed and the conclusions that can be drawn from internal evaluation alone. A systematic review of public glaucoma databases by Da Silva et al. [9] identified recurring structural limitations: small sample sizes relative to the complexity of the classification problem, unbalanced class distributions reflecting real-world glaucoma prevalence, inconsistent annotation standards across institutions, and a lack of accompanying clinical metadata such as intraocular pressure, corneal thickness, or visual field indices. These limitations mean that a model achieving strong accuracy on one dataset may be responding to source-specific image characteristics rather than to the pathological signal underlying glaucoma.

Among the most widely used benchmarks, RIM-ONE and DRISHTI-GS have appeared in the majority of comparative studies, often in isolation. Zedan et al. [3] found DRISHTI-GS1 and RIM-ONE to be the dominant benchmarks across 52 deep-learning studies reviewed, while Coan et al. [2] similarly identified a concentration of evaluation effort on a small dataset pool. The ORIGA dataset, comprising approximately 650 images from the Singapore Malay Eye Study and frequently used for optic disc and cup segmentation benchmarking, introduces a different acquisition domain and population proposed a CNN-based framework for automated classification of peripapillary atrophy from fundus images, which demonstrated that region-specific structural changes surrounding the optic disc can be used as discriminative factors to screen glaucoma. Their results highlight the potential for peripapillary features as additional biomarkers to cup-to-disc ratio estimation.

The more recent Hillel Yaffe Glaucoma Dataset (HYGD) [17], released through PhysioNet, adds 747 annotated deep fundus images with structured quality scores, offering a newer cohort with explicit quality grading that enables quality-stratified evaluation. The heterogeneity in image resolution, field of view, camera characteristics, acquisition protocol, disease prevalence, and annotation culture across these four datasets makes them a particularly informative set for evaluating cross-dataset robustness. This is the core rationale for the four-dataset benchmark design of the present study.

### 2.2. Methodological Landscape of Fundus-Based AI for Glaucoma Screening

Two complementary systematic reviews provide a useful taxonomy of the methodological landscape. Coan et al. [2] analysed 36 publications from 2011 to 2021 and distinguished one-step end-to-end classifiers from two-step frameworks that first segment the optic disc and cup before performing binary classification, noting that two-step approaches offer interpretability benefits aligned with clinical transparency requirements. Among the machine-learning frameworks reviewed, SVMs were the most common classifier, and a linear mixed-effects model using a 24-point profile cup-to-disc ratio achieved an AUROC of 0.997 on internal validation and 0.969 externally. Zedan et al. [3] extended this picture by reviewing 52 deep-learning studies from 2019 to 2023, finding ResNet, VGG, InceptionNet, EfficientNet, and U-Net variants to be the most prevalent architectures, and identifying low contrast between the optic cup and adjacent disc tissue and artefact introduction by image enhancement algorithms as recurring technical limitations.

Hybrid frameworks combining deep and handcrafted features have shown persistent competitiveness under limited-data conditions. Mahum et al. [12] proposed a hybrid architecture jointly extracting CNN features alongside Histogram of Oriented Gradients (HOGs), Local Binary Pattern (LBP), and Speeded-Up Robust Features (SURFs) descriptors applying minimum-redundancy maximum-relevance feature selection, and classifying with a Random Forest, achieving 99% accuracy on DRISHTI-GS and RIM-ONE. This result illustrates how handcrafted representations can complement learned features when labelled sets are small, a condition that is common in clinical ophthalmic imaging where expert annotation is expensive. Transfer learning has been pursued as a natural response to annotation scarcity. Govindan et al. [14] compared AlexNet, VGG16, ResNet50, and InceptionV3 across ORIGA, STARE, and REFUGE, finding that a hybrid ResNet50–InceptionV3 combination achieved F1 scores up to 99.2%. Alghamdi et al. [10] evaluated a systematic family of VGG-16-based methods on RIM-ONE and RIGA: a transfer CNN (TCNN) fine-tuned on target data, a semi-supervised extension (SSCNN) that iteratively assigned pseudo-labels to high-confidence unlabelled samples, and an SSCNN-DAE variant that added a convolutional denoising autoencoder for unsupervised pre-training. The SSCNN-DAE achieved 93.8% accuracy and 95% AUC on RIM-ONE, illustrating how semi-supervised models can exploit the large volumes of unannotated retinal images available in clinical repositories.

Statistical rigour as an independent design criterion was foregrounded by Song et al. [15], who combined Retinex-based illumination normalisation with a compact four-layer CNN and systematic Design of Experiments hyperparameter optimisation, achieving 0.97 accuracy with standard errors below 0.03. This work reinforces the principle that reproducibility and rigorous tuning are at least as important as architectural sophistication for establishing clinically credible results. At the deployable end of the complexity spectrum, Sidhu et al. [18] trained a ResNet50V2 model on 1375 fundus photographs, obtaining 81.3% accuracy and 83% sensitivity, and positioning the model explicitly as a teleophthalmology adjunct rather than a standalone diagnostic tool, exemplifying the enduring trade-off between architectural complexity and deployment practicality.

Segmentation-integrated and attention-based architectures have further extended this methodological landscape, combining optic disc/cup segmentation, ensemble classification, or statistical texture descriptors with classification pipelines [13,19,20], and more recently incorporating attention mechanisms and multimodal fusion with OCT to achieve strong internal accuracy [21,22]. For example, Okuwobi et al. [22] presented Glauco-Net, achieving an AUC-ROC of 0.989 on REFUGE across six datasets. While these results illustrate the representational capacity achievable under favorable internal conditions, none of the reviewed studies systematically evaluate cross-dataset generalisation, calibration, or threshold stability, which motivates the present benchmark’s focus on these dimensions.

Table 1 summarises the representative studies selected to contextualise the deployment-oriented scope of the present benchmark. The studies were selected for the following reasons: (i) relevance to glaucoma detection or screening using retinal imaging; (ii) fundus photography and/or related ophthalmic techniques such as OCT, OCT angiography, visual fields, or clinical metadata; (iii) use of modern machine learning or deep learning techniques (CNNs, transformers, attention mechanisms, multimodal models, domain-adaptation techniques, semi-supervised/self-supervised learning, hybrid handcrafted–deep feature pipelines, etc.); (iv) emphasis on external validation, domain shift, interpretability, or deployment-related evaluation when available; and (v) publication recency (preferably 2024–2026). The previous studies were only considered if they are relevant to the model families under consideration in this benchmark (e.g., semi-supervised CNNs or hybrid deep-handcrafted models). This table is not an exhaustive systematic review but rather a well-organized comparison of the representative works to what is being considered in the evaluation section.

### 2.3. Recent Transformer, Attention, and Multimodal Approaches

Glaucoma-AI research has advanced significantly in the last few years, from conventional convolutional neural networks to transformer-based, attention-guided, multimodal, explainable, semi-supervised, self-supervised, domain-adaptive, and hybrid deep-handcrafted architectures [23,27,29,30,31].

One of the most significant challenges in the recent literature is cross-dataset generalisation. Models trained on one dataset, for example, can perform well inside them, but do not perform well on images from other cameras, populations, institutions, or annotation protocols. To this end, domain adaptation and domain generalisation studies have been done to learn representations that do not depend so much on source-specific image characteristics. Madadi et al. [8] proposed a glaucoma domain-adaptation framework to improve data transfer across many datasets. This work is of great interest because it clearly states that domain shift is a major problem and can be a barrier to clinical use. However, many domain-adaptation methods need some access to the target domain in training or adaptation, while in this study we evaluate fully zero-shot cross-dataset transfer, where the target datasets are not used in training, validation, threshold selection, or recalibration.

Transformer-based models have also become more popular as self-attention mechanisms can capture long-range spatial dependencies in retinal images and may be able to help to explain optic-disc, optic-cup, neuroretinal-rim, retinal nerve fiber layer, and peripapillary structural changes associated with glaucoma [23,24,25]. Attention-guided CNNs and hybrid CNN–transformer architectures have also been proposed to focus on clinically relevant regions in order to improve representation learning in fundus-based glaucoma screening [23,25]. While these models are known to be highly discriminating under internal assessment, they are often ranked on accuracy, sensitivity, specificity, or AUC and less reliable in the way of probability calibration, threshold portability, repeated-seed variability, and computational cost.

Multimodal glaucoma-AI systems have attempted to combine complementary information from fundus photographs, OCT, OCT angiography, visual fields, or clinical metadata [23,26,32]. Such approaches are attractive from a clinical point of view as glaucoma is a multifactorial disease and a diagnosis is rarely made based on one imaging modality. However, multimodal systems can have other drawbacks that are difficult to deploy; for instance, these can be missing modalities in the system, heterogeneous acquisition protocols are needed, cost is higher, and portability of the system is limited in the clinical setting [26,32] so fundus-only screening is still of value in low-resource and scalable settings to ensure that its limitations under domain change are understood.

Semi-supervised and self-supervised learning approaches are also important in ophthalmic AI as expert-labelled glaucoma images are usually small and sparse, but unlabelled retinal images are more prevalent. Semi-supervised CNNs, pseudo-labelling strategies, and denoising-autoencoder pretraining have been employed to exploit low-labelled fundus images and improve representation learning under limited-label conditions. The more recent self-supervised and foundation-model approaches in ophthalmology aim to learn transferable retinal representations from large-scale unlabelled image collections. However, more detailed feature representation is not enough to ensure good probability estimates, stable operating thresholds, or robust zero-shot performance when dataset change is used in the future, and hence we have included transfer-learning and semi-supervised versions of VGG16 in this benchmark, as well as explicit calibration and cross-dataset evaluation.

In our experience, hybrid deep-handcrafted approaches remain relevant with the rise of end-to-end deep learning. These methods combine the learned CNN features with handcrafted descriptors such as HOG, LBP, SURF, annular-region features, or texture statistics and then use classical classifiers or feature selection methods [12,25]. Such methods may be useful in the context of limited data modeling as a handcrafted gradient and texture descriptor can better encode structural information that is not tied to the dataset-specific colour, contrast, or illumination statistics. However, previous hybrid studies have primarily focused on the internal classification performance.

In addition, explainable-AI methods have become more important in glaucoma detection because clinical adoption will require high discrimination but also transparent and anatomically plausible decision support [27,28]. Saliency maps, Grad-CAM visualisations, attention maps, and concept-based explanations have been used to assess if models focus on clinically meaningful structures including the optic disc, optic cup, neuroretinal rim, retinal nerve fiber layer defects, and peripapillary atrophy [27,28]. However, explainability alone is not enough to guarantee robustness: models with plausible heatmaps can still be poorly calibrated or can degrade substantially under domain shift [8,30].

Together, this 2024–2026 literature shows some rapid progress in research but also shows that there is still a long-standing gap in the field of evaluation. There have been several studies on strong model architectures, domain-adaptation strategies, transformer-based and attention-guided models, multimodal systems, explainable-AI frameworks, semi-supervised learning, self-supervised representation learning, and hybrid deep-handcrafted approaches. We were not aware of a fundus-based glaucoma screening benchmark with four heterogeneous public datasets, multiple model families, preprocessing ablation, random seeds, probability calibration, threshold stability, computational profiling, statistical testing, and extensive zero-shot cross-dataset transfer. This gap is the motivation for our benchmark, which evaluates model reliability not only by internal discrimination but also by external robustness, calibration, threshold portability, reproducibility, and practical deployability.

### 2.4. External Validation, Domain Shift, and Calibration

Despite the impressive internal results reported in many glaucoma-AI studies, a consistent and underexamined problem is that models often perform markedly worse when evaluated on data from a different acquisition source, population, or clinical protocol. It is also worth considering how our hybrid framework is compared with more recent hybrid architectures with metaheuristic optimisation, explainable AI overlays and attention-augmented feature weighting. Such hybrid frameworks tend to report good internal performance by further improving feature selection or feature weighting but few report cross-dataset transfer, calibration behaviour or threshold stability. Framework 1 in our benchmark has a relatively simple CNN + HOG + LBP + mRMR + Random Forest pipeline without any metaheuristic optimisation in place. This allows the benchmark to test a handcrafted–deep feature fusion pipeline under both within-dataset and cross-dataset conditions. However, any robustness advantage should still be viewed as the full CNN + HOG + LBP + mRMR + RF configuration and not as feature fusion alone. Extending the benchmark to include attention-augmented or optimisation-enhanced hybrid variants is a natural direction for future work.

Madadi et al. [8] developed the Glaucoma Domain Adaptation (GDA) model, which uses low-rank representation and a progressive weighting mechanism to separate domain-invariant from domain-specific features, demonstrating improved generalisation across the OHTS, ACRIMA, and RIM-ONE datasets. Their results underline that domain adaptation is not merely a technical refinement but a prerequisite for generalisable glaucoma screening. Despite such advances, domain adaptation methods typically require some degree of target-domain access during training, and their performance under fully blind transfer to unseen acquisition domains remains an open research question. This limitation motivates evaluation protocols that include explicit, fully held-out cross-dataset transfer as a primary assessment criterion rather than a secondary experiment.

Calibration is a closely related but distinct aspect of model quality that receives insufficient attention in the glaucoma-screening literature. A well-calibrated classifier produces probability estimates that accurately reflect empirical outcome frequencies; for example, a model that assigns a predicted glaucoma probability of 0.8 to a set of images should have approximately 80% of those images truly glaucomatous [7]. Poor calibration can arise independently of discrimination performance: a model can achieve high AUC while systematically overestimating or underestimating confidence, which is especially problematic in a clinical triage context where the output probability informs referral decisions. Yet most glaucoma-AI studies report only discrimination metrics such as accuracy, sensitivity, specificity, and AUC, without measuring Brier score, expected calibration error, or the stability of operating thresholds across settings. The present study directly addresses this gap by treating calibration and threshold stability as first-class evaluation criteria alongside standard discrimination metrics.

### 2.5. Fundus Photography Versus Optical Coherence Tomography: Clinical and Algorithmic Implications

The choice of imaging modality is both a clinical and an algorithmic decision, and understanding the trade-offs between fundus photography and optical coherence tomography (OCT) is important for contextualising the strengths and limitations of fundus-based AI benchmarks. Beniz et al. [5] conducted a comprehensive review of both modalities in the context of glaucoma screening, finding that OCT is highly accurate and reproducible due to its objective, quantitative measurement of retinal nerve fibre layer thickness, but that its cost, lower portability, and requirement for specialist infrastructure limit its suitability for large-scale population screening. Colour fundus photography, by contrast, is more accessible and cost-effective, though its diagnostic value depends critically on image quality, illumination consistency, and the reliability of grading, all of which introduce algorithmic challenges not present in quantitative OCT measurements.

From an optical and signal-quality perspective, fundus photographs encode retinal structure through reflected broadband light, making them sensitive to non-pathological sources of variation including pupil dilation, camera focus, exposure settings, operator technique, and media opacities such as early cataract. These sources of variation produce systematic differences between datasets acquired under different protocols and thus contribute directly to the domain-shift problem described in the previous subsection. The practical implication for fundus-based AI benchmarking is that preprocessing strategies intended to reduce illumination non-uniformity, such as Retinex-based normalisation, or to restrict analysis to the diagnostically most informative region, such as region-of-interest extraction, may improve or harm performance depending on the acquisition characteristics of the specific dataset at hand. Evaluating preprocessing strategies under both within-dataset and cross-dataset conditions, as the present benchmark does, is therefore important for understanding which interventions are genuinely robust versus which are overfitted to a particular acquisition domain.

The growing literature on AI-enhanced fundus-photograph analysis reviewed by Cao et al. [6] further illustrates that, despite the optical limitations of the modality, fundus-based approaches remain an important focus for population-level glaucoma screening because of their cost-accessibility and the global distribution of non-mydriatic fundus cameras in primary-care settings. This motivates continued work on making fundus-based AI more robust, better calibrated, and more reliably deployable rather than assuming that OCT or multimodal pipelines will displace fundus-based screening in the near term.

### 2.6. Synthesis and Motivation for the Present Benchmark

The expanded literature review shows that glaucoma-AI research has made significant progress in discriminating glaucomatous from healthy fundus images under internally matched evaluation conditions. In recent years, transformer-based networks, attention mechanisms, multimodal fusion, domain-adaptation strategies, explainable AI methods, and hybrid deep-handcrafted feature pipelines have been proposed. These developments demonstrate the technical maturity of the field. However, they also show an important gap: Most studies continue to focus on internal accuracy, sensitivity, specificity, or AUC while deployment-related dimensions such as cross-dataset transportability, probability calibration, threshold stability, repeated-seed variability, preprocessing sensitivity, and computational cost are rarely explored together.

This distinction is really the key to the novelty of this paper. We do not claim that cross-dataset evaluation, calibration analysis, semi-supervised learning, hybrid feature fusion, or preprocessing assessment are new. Rather, we have combined them in a single, controlled benchmark. We have evaluated six model implementations on four heterogeneous public datasets, four preprocessing strategies, five random seeds, validation of MCC threshold selection, Brier score and ECE calibration, threshold stability assessment, computational cost profiling, non-parametric statistical testing, and twelve zero-shot cross-dataset transfer tasks.

This integrated approach addresses a deployment-oriented question that is not adequately answered in the literature: which class of model remains most reliable when we do not use our internal evaluation, but rather use a system (as we did in the present paper) to test against the external background. In this work, reliability is not only measured by discrimination performance, but also by external robustness, probability calibration, threshold portability, seed-level reproducibility, preprocessing sensitivity, and computational feasibility. The experimental procedure is described in the following sections and comparative evidence is presented.

## 3. Methodology

### 3.1. Overview

This study implements a controlled, reproducible benchmark for automated glaucoma screening from retinal fundus images, evaluating six model implementations spanning three methodological families across four public datasets: RIM-ONE, DRISHTI-GS, HYGD, and ORIGA. The compared families consist of a hybrid deep–handcrafted random-forest framework, a transfer-learning and semi-supervised VGG16 family, and a compact Retinex-enhanced convolutional neural network. All methods are evaluated under a unified experimental protocol comprising repeated experiments over five random seeds, explicit preprocessing ablation across four input configurations, balanced training, validation-based threshold optimisation, test-time augmentation (TTA), calibration analysis, within-dataset testing, and exhaustive ordered cross-dataset transfer evaluation.

**Figure 1:**
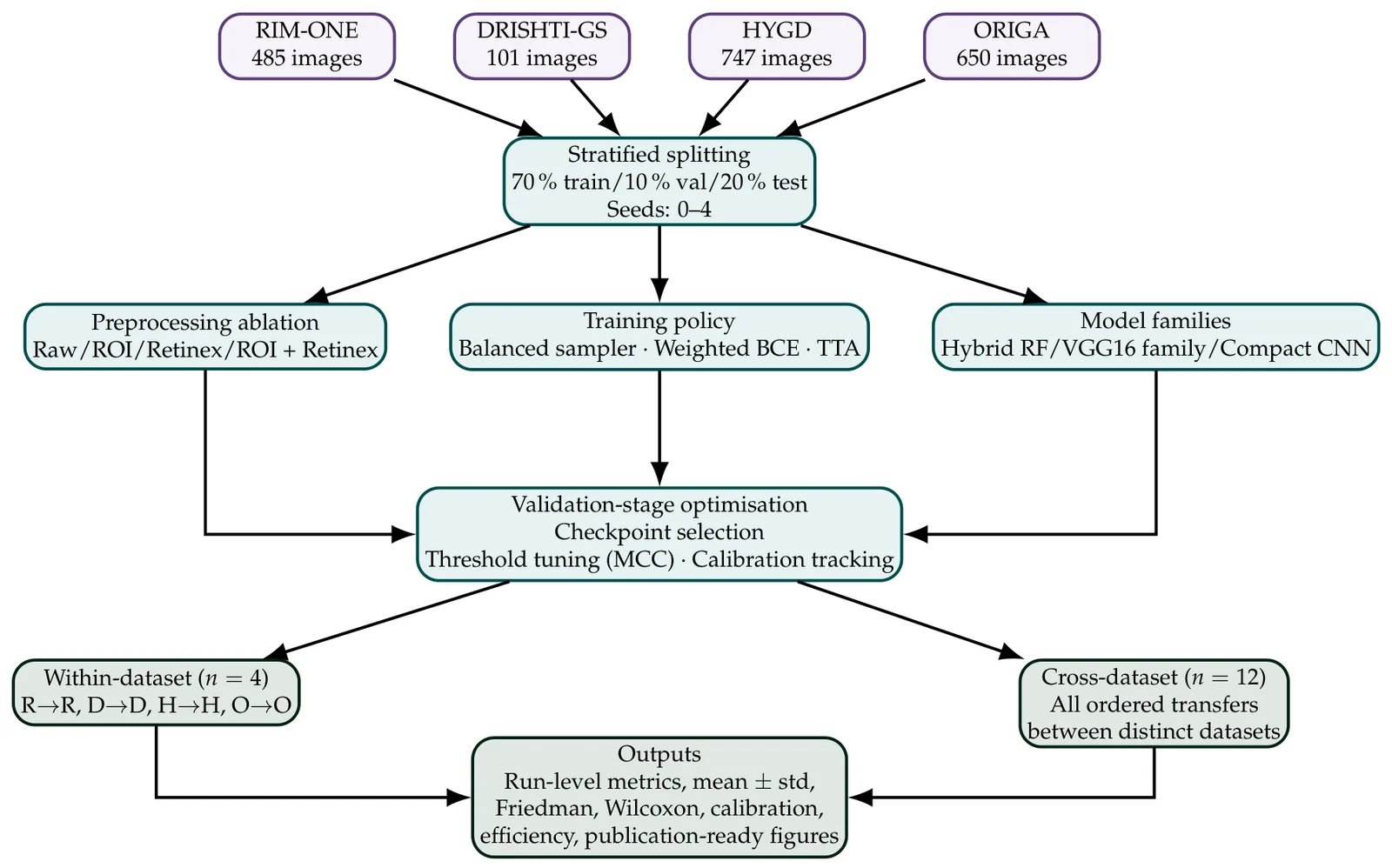
Overall benchmark pipeline. Models are trained and validated on one dataset and evaluated both on the same dataset (within) and on held-out test sets from the remaining datasets (cross). R = RIM-ONE; D = DRISHTI-GS; H = HYGD; O = ORIGA.

The four-dataset design was chosen deliberately to improve both internal validity and external relevance. A four-dataset benchmark comprising both within-dataset and cross-dataset evaluation generates four within-dataset tasks and twelve ordered cross-dataset transfer tasks, thereby enabling each model family to be examined not only under in-domain conditions but also under the kind of acquisition heterogeneity that characterises real-world deployment. By combining datasets with substantially different image appearance, disease prevalence, annotation conventions, and acquisition origin, the benchmark avoids the common limitation of single-source evaluation while remaining grounded in publicly available and reproducible data [9]. The overall experimental pipeline is summarised in Figure 1.

#### Experimental Design at a Glance

Given the number of datasets, model families, preprocessing configurations, random seeds, and evaluation tasks involved in this benchmark, Table 2 summarizes the overall experimental design in a single consolidated view before the detailed description of datasets, preprocessing, and model families that follows in the subsequent subsections.

### 3.2. Datasets

The benchmark uses four public retinal fundus image datasets that differ substantially in size, class balance, acquisition origin, and annotation protocol. This heterogeneity was a primary selection criterion: a benchmark intended to examine cross-dataset robustness should not draw from datasets produced under similar conditions, as this would underestimate the domain shift that models face in practical deployment [8,9].

**Figure 2:**
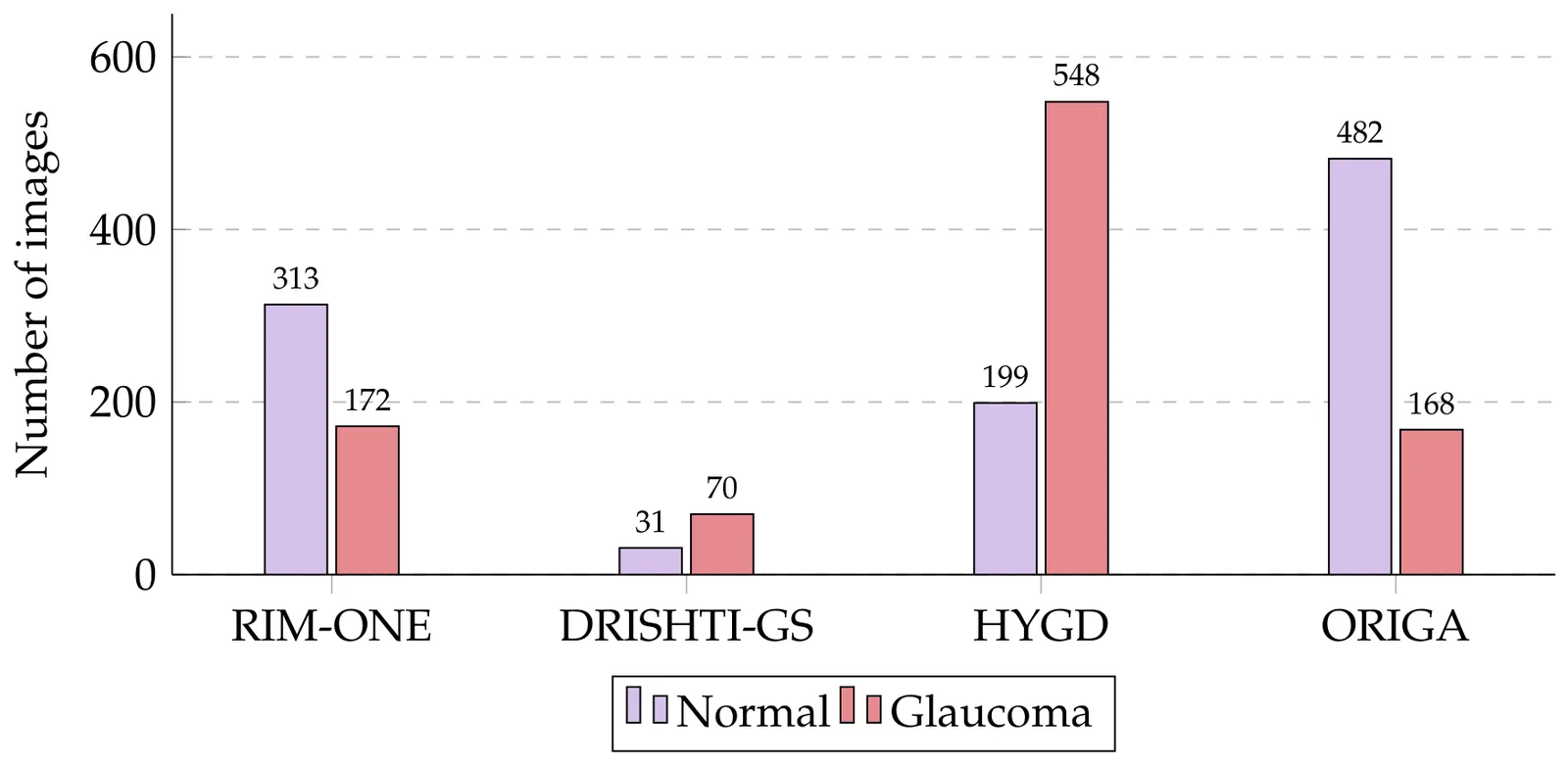
Class distribution (normal versus glaucoma) across the four benchmark datasets, corresponding to the counts reported in Table 3. RIM-ONE and ORIGA are normal-majority cohorts, whereas DRISHTI-GS and HYGD are glaucoma-majority cohorts, illustrating the inverted prevalence structure that motivates the cross-dataset stress-test design of this benchmark.

**Figure 3:**
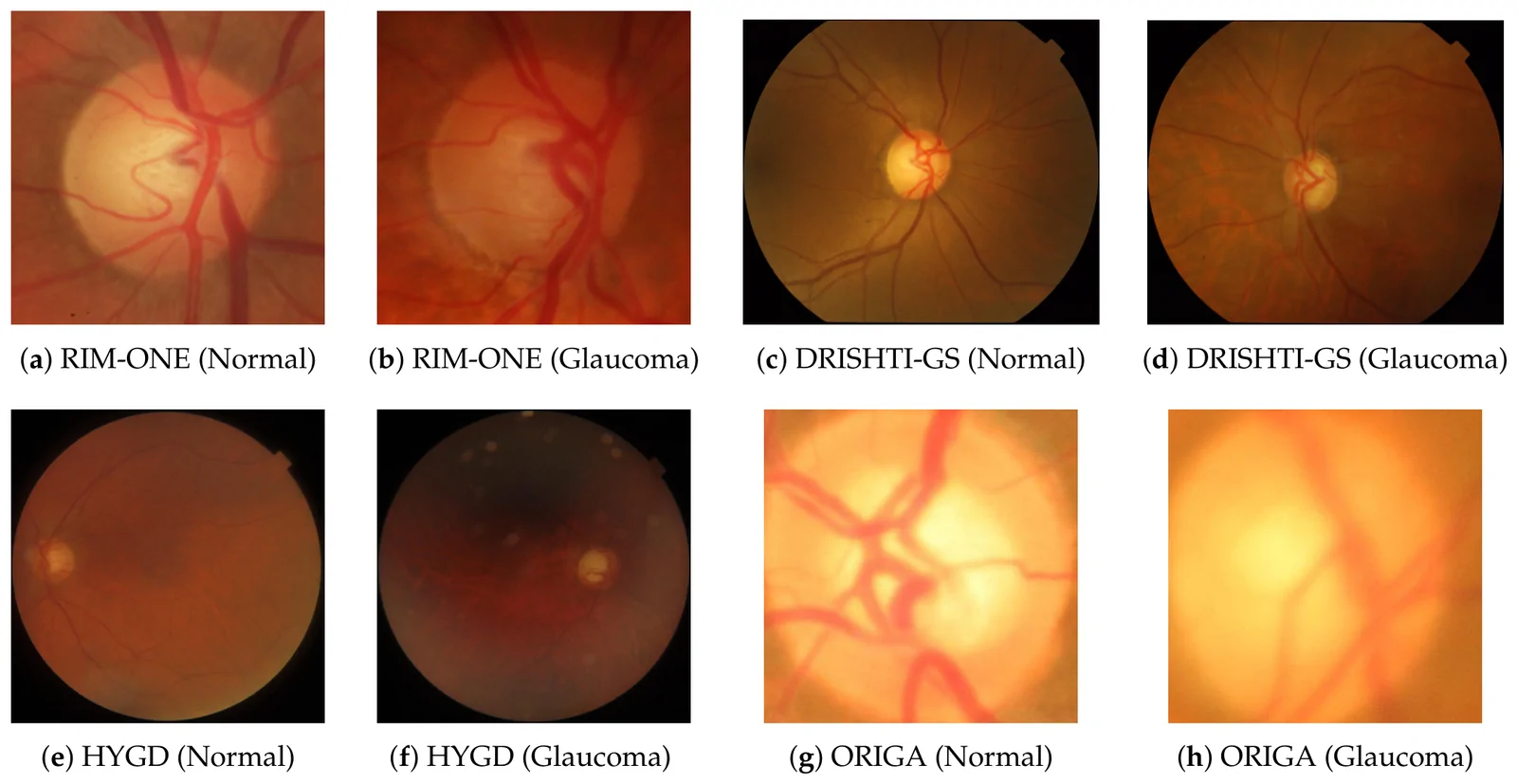
Representative normal and glaucomatous fundus images from each of the four benchmark datasets prior to preprocessing: (**a**,**b**) RIM-ONE, (**c**,**d**) DRISHTI-GS, (**e**,**f**) HYGD, and (**g**,**h**) ORIGA. Differences in field of view, colour balance, optic-disc framing, and image resolution are visible across cohorts, illustrating the acquisition heterogeneity underlying the cross-dataset transfer challenge examined in this study.

Figure 2 presents the class distribution (normal versus glaucoma) for each of the four datasets, corresponding to the counts reported in Table 3, and Figure 3 shows representative normal and glaucomatous example images from each dataset prior to preprocessing, illustrating the heterogeneity in image appearance, field of view, and disease presentation across cohorts.

#### 3.2.1. RIM-ONE

RIM-ONE [33] is one of the most widely adopted publicly available fundus image datasets in the glaucoma-screening literature, having been used in 22 of the 52 studies surveyed by Zedan et al. [3], which ensures direct comparability with a broad body of prior work. It comprises 485 colour fundus images, including 313 normal and 172 glaucomatous cases. Its relatively large size and moderate class imbalance (approximately 65% normal versus 35% glaucomatous) provide a statistically stable evaluation environment for the repeated-seed protocol while also reflecting a realistic screening prevalence profile. The images were acquired under comparatively consistent clinical conditions, making RIM-ONE appropriate for establishing within-dataset discriminative baselines before introducing the additional challenge of cross-dataset transfer.

#### 3.2.2. DRISHTI-GS

DRISHTI-GS [34] was selected to complement RIM-ONE along dimensions that reveal the fragility of models trained under narrow distributional conditions. It comprises 101 images with 70 glaucomatous and 31 normal cases, exhibiting an inverted class distribution relative to RIM-ONE. This inversion is clinically important for cross-dataset transfer: a model transferred from a normal-majority source to a glaucoma-majority target must contend with both an acquisition-domain mismatch and a substantial prior probability shift, constituting a more demanding and realistic stress test than two datasets with similar class ratios would provide [2,3]. DRISHTI-GS appeared in 24 of the same 52 studies reviewed by Zedan et al. [3], confirming its standing as a standard reference alongside RIM-ONE. Its smaller size also stress-tests model stability under limited-data conditions, providing a complementary evaluation environment to the larger corpora.

#### 3.2.3. HYGD

The Hillel Yaffe Glaucoma Dataset (HYGD) [17] was included to extend the benchmark with a newer, clinically annotated cohort that explicitly addresses a known limitation of many existing fundus datasets: the absence of gold-standard annotation and acquisition quality metadata. In the PhysioNet release used here, HYGD contains 747 deep fundus images annotated for glaucomatous optic neuropathy, comprising 548 glaucoma-positive and 199 glaucoma-negative cases, together with a structured Labels.csv providing image name, patient identifier, binary diagnostic label, and a per-image quality score on a 1–10 scale. The explicit quality scoring makes HYGD particularly valuable in a multi-dataset benchmark because it enables quality-stratified analysis and exposes potential interactions between image quality and model robustness [17]. The marked class imbalance (73.4% glaucomatous) also differs from that of the other three datasets, introducing an additional axis of distributional heterogeneity. In the present pipeline, the HYGD labels GON+ and GON- were mapped to glaucoma and normal, respectively.

#### 3.2.4. ORIGA

ORIGA [35] was selected to introduce a further distinct acquisition domain and a different epidemiological profile. The dataset comprises approximately 650 fundus images, including 482 normal and 168 glaucomatous cases, acquired as part of the Singapore Malay Eye Study. With a glaucoma prevalence of approximately 25.8%, ORIGA exhibits a lower disease fraction than HYGD and DRISHTI-GS, contributing a third distinct prior-probability profile to the benchmark. ORIGA has served as a standard reference dataset in the glaucoma-AI literature and has been used in studies of optic disc and cup segmentation and supervised classification, making its results directly comparable to prior work [6,9]. Its different population characteristics, acquisition equipment, and image processing pipeline relative to RIM-ONE, DRISHTI-GS, and HYGD make it an informative cross-dataset target for evaluating whether model rankings remain stable across heterogeneous sources.

It is important to mention that across the four datasets, demographic variables were either not included in the released benchmarking files or not usable as structured screening inputs. DRISHTI-GS was collected at Aravind Eye Hospital in Madurai, India, from patients aged 40–80 years with approximately balanced sex distribution, as described in the original publication; however, the public dataset release provides only optic disc/cup segmentations, soft maps, and cup-to-disc ratio values, without individual-level age or race/ethnicity fields in the downloadable data structure [34]. RIM-ONE, originally introduced by Fumero et al., comprises stereo retinal fundus images acquired in Spain and later expanded in the RIM-ONE DL version; both releases provide image-level glaucoma labels and segmentation masks but do not include structured demographic variables such as age, sex, or race/ethnicity in the distributed files [33]. The HYGD dataset (PhysioNet release) reports an aggregate age range (36–95 years) and balanced sex distribution in its descriptive documentation, but individual-level age, race/ethnicity, and broader clinical history are not included in the core benchmarking metadata, and the cohort originates from a single geographic location [17]. ORIGA-light was derived from the Singapore Malay Eye Study, which sampled Malay adults aged 40–79 years; consequently, the cohort is ethnically homogeneous by design, and the released image dataset used for artificial intelligence benchmarking includes segmentation masks and glaucoma labels but not structured individual demographic variables such as age [35,36]. Therefore, although aggregate demographic characteristics are described in the source publications, race and age were not available as structured, model-level inputs and were not incorporated into the screening models evaluated in this study.

#### 3.2.5. Dataset Harmonisation and Label Standardisation

Although the four datasets share the same binary classification objective, they originate from different sources and use heterogeneous file layouts, directory structures, and metadata conventions. A unified discovery and harmonisation step was therefore introduced before training. All labels were mapped to a common binary formulation in which class 0 denotes normal and class 1 denotes glaucoma. For HYGD, this mapping was applied directly to the GON-/GON+ labels in Labels.csv. For ORIGA, the preparation pipeline supports class-folder layouts as well as archive-provided metadata tables. For RIM-ONE and DRISHTI-GS, folder structure and filename conventions were parsed programmatically.

This harmonisation step is important not only for code uniformity but also for statistical validity: if label encodings are inconsistent across datasets, cross-dataset transfer experiments would conflate genuine domain shift with trivial label-coding artefacts. Harmonisation ensures that all sixteen experimental tasks (four within-dataset, twelve cross-dataset) are performed on a consistent binary classification target and that any observed performance variation is attributable to distributional differences between datasets rather than to annotation inconsistency.

#### 3.2.6. Dataset Partitioning

Each dataset was partitioned using a stratified 70:10:20 train/validation/test split, and the same protocol was repeated independently for seeds 0–4 to quantify run-to-run variability. In the semi-supervised setting, the labelled fraction was fixed at 0.6 of the training partition, with the remainder treated as unlabelled during pseudo-label generation. For cross-dataset evaluation, a model trained and validated on one dataset’s split was tested directly on the held-out test split of a different dataset, without any target-domain fine-tuning. This zero-shot transfer protocol provides a clean measure of distributional robustness, because any information from the target domain is entirely excluded from training and threshold selection.

Table 3 reports the target split sizes for each dataset. Several methodological choices merit explicit justification. Five random seeds were selected as a pragmatic compromise between characterising run-to-run variability (Section 4.7) and the substantial computational cost of repeating all sixteen within- and cross-dataset tasks across four preprocessing configurations and six models. The 70:10:20 stratified split was adopted to preserve class-ratio stratification within each dataset, whose prevalence differs substantially across cohorts (Table 3), while retaining validation and test partitions of sufficient size for stable threshold tuning and metric estimation, even for the smallest dataset (DRISHTI-GS, 101 images). MCC was selected as the threshold-optimisation criterion, rather than accuracy or F1, because it incorporates all four confusion-matrix entries and remains informative under substantial class imbalance (Section 3.7). We did not employ k-fold or nested cross-validation because the central objective of this study is zero-shot cross-dataset transfer, which requires the target-domain test set to remain entirely untouched during source-domain training, validation, and threshold selection; repeated within-dataset k-fold splitting would not alter this requirement while substantially increasing computational cost. We acknowledge, however, that nested cross-validation combined with cross-dataset transfer represents a valuable, more computationally intensive extension for future work.

#### 3.2.7. Data Augmentation

**Figure 4:**
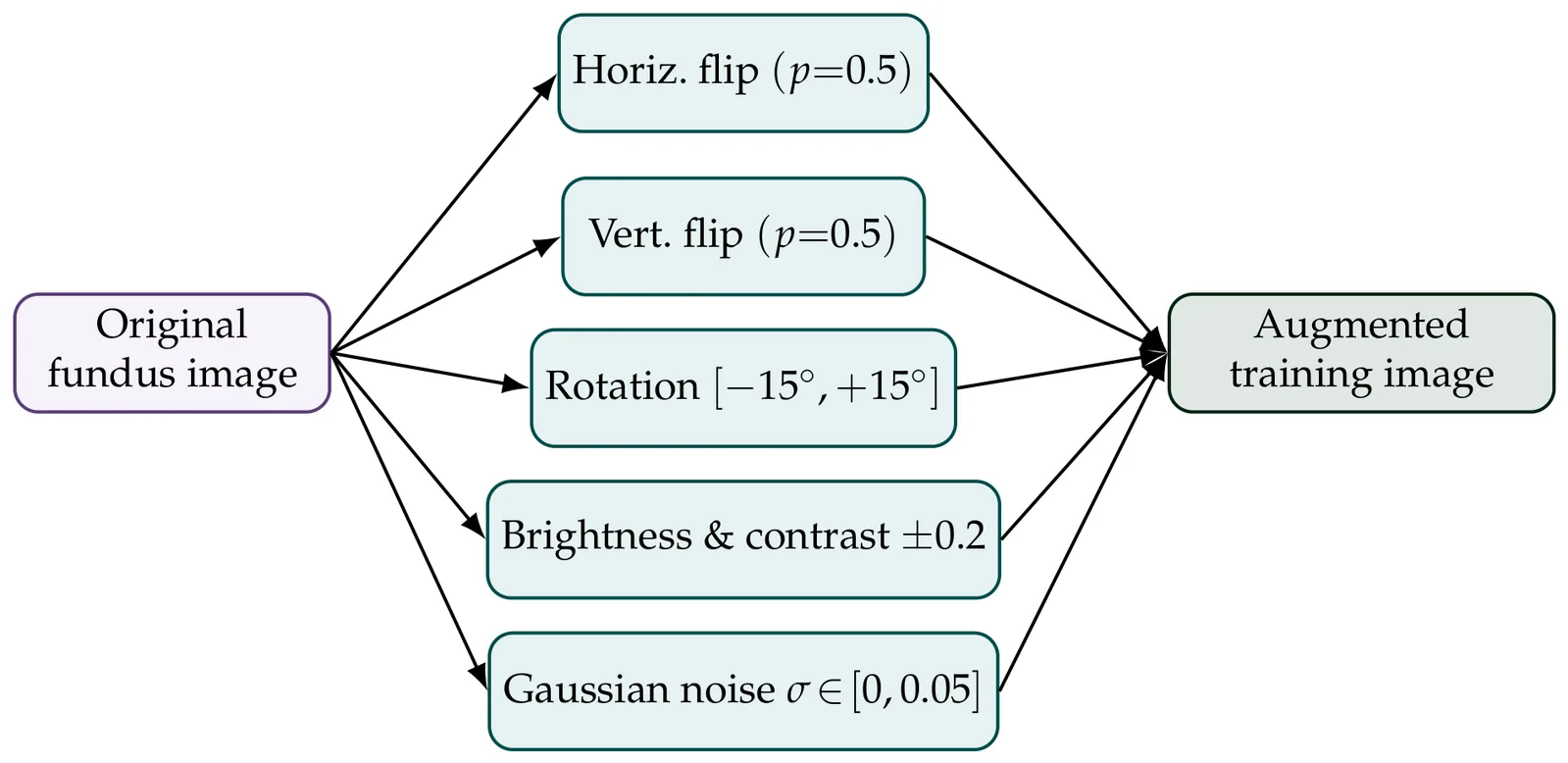
Stochastic data augmentation pipeline applied uniformly to all compared deep-learning frameworks during training. Each transformation is sampled independently at every training step.

All deep-learning frameworks were trained with the same stochastic augmentation policy, applied only to the training partition to avoid contaminating validation or test performance. The policy consists of random horizontal flipping (p=0.5), random vertical flipping (p=0.5), random rotation within [−15°,+15°], random brightness adjustment within ±0.2, random contrast adjustment within ±0.2, and additive Gaussian noise with standard deviation sampled uniformly from [0,0.05]. Applying the same policy to all frameworks ensures that observed performance differences reflect modelling strategy rather than unequal regularisation. The augmentation pipeline is illustrated schematically in Figure 4, where each transformation is sampled stochastically and independently at every training step.

### 3.3. Preprocessing

The preprocessing stage was implemented as an explicit ablation rather than a fixed assumption. Four input configurations were evaluated: *raw*, *ROI*, *Retinex*, and *ROI + Retinex*. This design enables the benchmark to determine whether optic-disc localisation and illumination normalisation provide consistent benefit across datasets and model families, or whether their effects are source-dependent.

The motivation for treating preprocessing as an ablation variable is grounded in the optical properties of fundus photography. Colour fundus images are sensitive to acquisition-related sources of variation such as camera type, lens characteristics, illumination non-uniformity, field of view, and operator technique. These sources of variation are particularly pronounced when images originate from different clinical settings, as is the case in this benchmark. Preprocessing strategies that reduce illumination-related variability may therefore be more or less beneficial depending on whether the dominant source of performance limitation is acquisition heterogeneity or structural image content. Evaluating all four configurations under both within-dataset and cross-dataset conditions directly addresses this question [5].

In the ROI-based configurations, the optic disc region was cropped to reduce background variability and focus the model on the anatomical region most strongly associated with glaucomatous structural change. All images were subsequently resized to 224×224 pixels to ensure a common input dimensionality.

For illumination normalisation, Multi-Scale Retinex with Colour Restoration (MSRCR) was applied after resizing. The single-scale Retinex response is defined as

(1) $$r_{s}\left(x,y\right)=\log I\left(x,y\right)-\log\;\left[F_{s}\left(x,y\right)*I\left(x,y\right)\right],$$

where $F_{s}$ is a Gaussian surround function with scale parameter *s*, and ∗ denotes convolution. The multi-scale response is computed as the weighted combination

(2) $$R\left(x,y\right)=\sum_{s\in S}w_{s}\;r_{s}\left(x,y\right),$$

with scales S={15,80,250} and uniform weights $w_{s}=\frac{1}{3}$. The three scales capture fine, medium, and coarse illumination gradients, respectively, enabling the normalisation to partially decouple the structural retinal content from acquisition-dependent illumination variation. This is especially relevant in multi-dataset benchmarking because it targets the same optical non-uniformity that differs systematically across acquisition domains.

**Figure 5:**
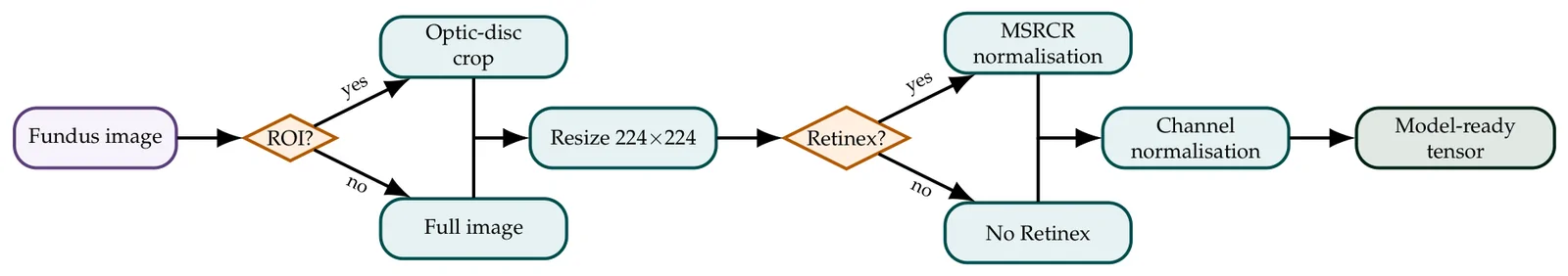
Standardised preprocessing workflow applied uniformly across all four datasets and all compared frameworks.

**Figure 6:**
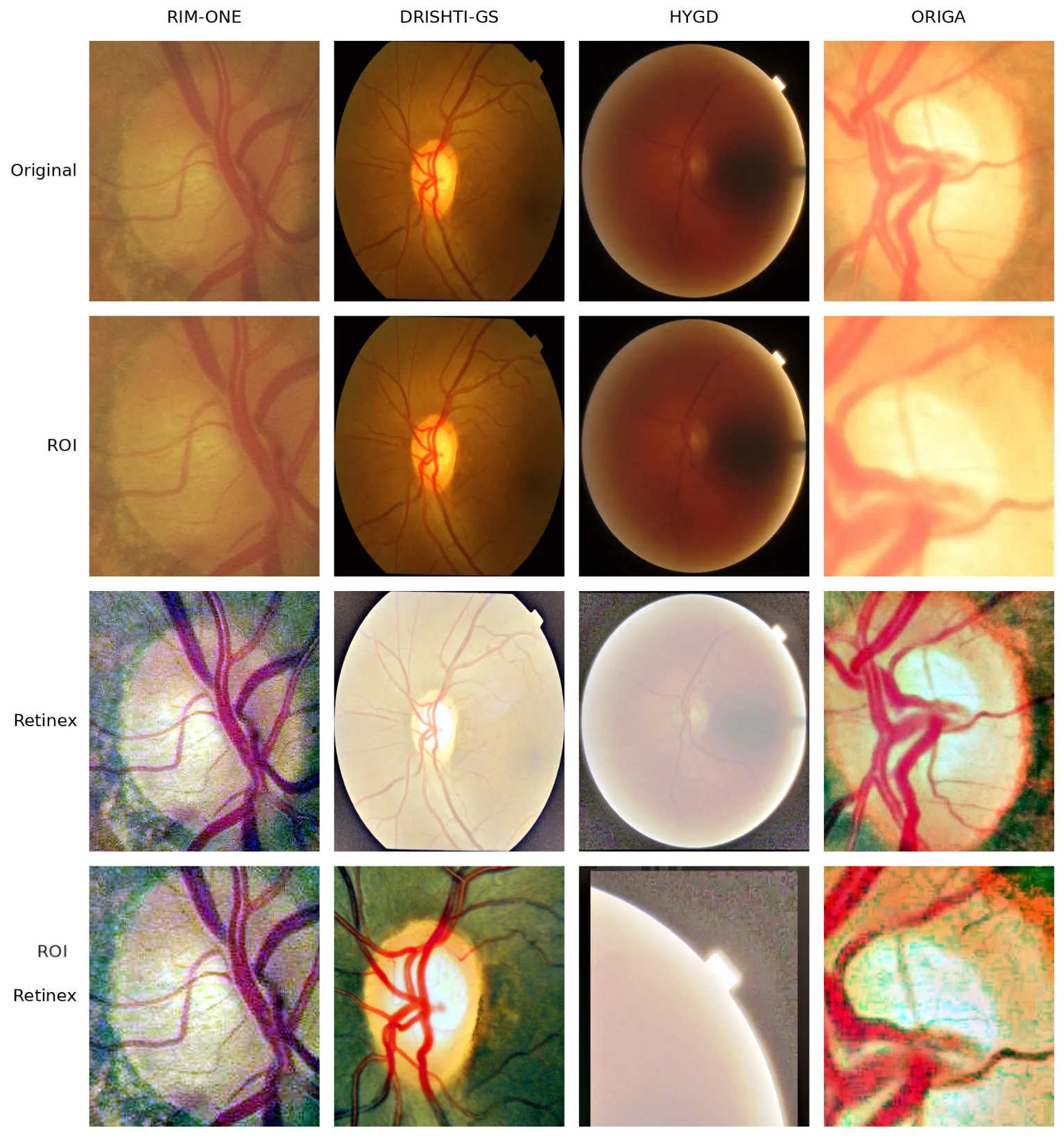
Representative preprocessing outputs for RIM-ONE, DRISHTI-GS, HYGD, and ORIGA. (**Top row**): original fundus images. (**Middle row**): region-of-interest (ROI) views. (**Bottom row**): ROI with Retinex normalisation. For HYGD and DRISHTI-GS, full images are retained after preprocessing; RIM-ONE and ORIGA remain optic disc–focused. All panels are resized for comparison.

Following resizing and optional Retinex enhancement, channel means and standard deviations computed on the training partition were used to normalise all splits. The same statistics were applied to validation and test images to prevent information leakage. The full preprocessing workflow is illustrated in Figure 5, and representative outputs of the roi and roi_retinex configuration are shown in Figure 6.

### 3.4. Framework Implementation

#### 3.4.1. Framework 1: Hybrid Deep–Handcrafted Random-Forest Model

**Figure 7:**
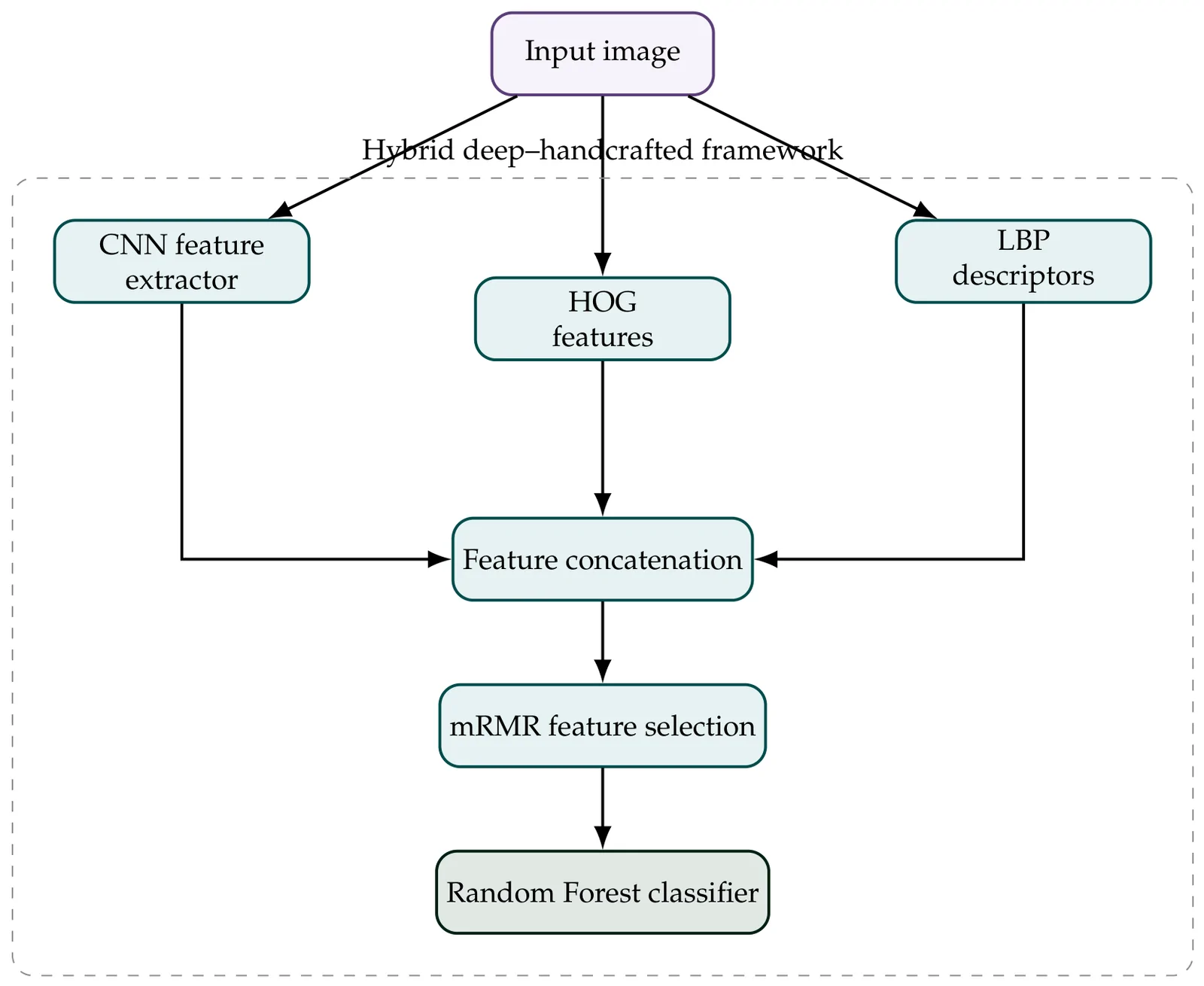
Architecture of the hybrid deep–handcrafted feature-fusion framework (Framework 1).

The first framework combines learned convolutional representations with handcrafted texture and local-pattern descriptors, fused before a Random Forest decision stage. This design follows the architecture introduced by Mahum et al. [12] and was retained because hybrid feature fusion has shown strong competitiveness under limited-data conditions and, in prior experiments, produced particularly stable cross-dataset performance. Its architecture is summarised in Figure 7.

Concretely, the hybrid feature-fusion model was implemented on the same 224×224 inputs used throughout the benchmark. The deep feature-extraction branch used a frozen ResNet-18 backbone with its final classification layer removed, yielding a 512-dimensional embedding for each image. This deep representation was concatenated with handcrafted descriptors computed from the same preprocessed image, including a Histogram of Oriented Gradients (HOGs) descriptor with 16×16 pixel cells, 2×2 cells per block, and 9 orientation bins (6084 dimensions), and a Local Binary Pattern (LBP) histogram using radius R=3 and p=24 neighboring points (26 dimensions). The resulting feature vector was standardized using training-partition statistics only, reduced through minimum-redundancy maximum-relevance (mRMR) feature selection, and classified using a class-balanced Random Forest. Feature selection follows the minimum-redundancy maximum-relevance (mRMR) principle: given a candidate feature set S and class label *c*, the selection objective is

(3) $$\max_{S}\left[\frac{1}{\left|S\right|}\sum_{f_{i}\in S}I\left(f_{i};c\right)-\frac{1}{{\left|S\right|}^{2}}\sum_{f_{i},f_{j}\in S}I\left(f_{i};f_{j}\right)\right],$$

where I(·;·) denotes mutual information. This criterion jointly maximises the relevance of selected features to the class label and minimises redundancy among them, producing a compact representation particularly well suited to small ophthalmic datasets. Applying mRMR to the 6622-dimensional fused feature vector retained the top 1324 features (approximately 20.0% of the original feature space) for classification, based on a fixed retention threshold applied within the training partition only.

Random Forest was selected as the decision-stage classifier for this hybrid feature pipeline due to its robustness in handling heterogeneous tabular feature vectors generated by combining CNN-based embeddings, HOG descriptors, and LBP histograms. As a comparison with a single decision tree, Random Forest reduces variance by bootstrap aggregation and random feature subspace sampling, requires relatively limited hyperparameter tuning, and is computationally efficient for the reduced mRMR feature space. It is also less sensitive to feature scaling and multicollinearity than many single model classifiers, which is an important feature to consider when mixing deep and handcrafted descriptors. Random Forest also provides feature-importance estimates that are in line with the interpretability goals of the benchmark.

The number of features considered was set to 1324 since this is 20% of the original 6622-dimensional fused feature vector. We set this high retention value to reduce redundancy and computational complexity while maintaining the diversity of data sources from the CNN, HOG, and LBP. The mRMR was used in the training component to avoid the leakage of information. Thus, the 1324 features we selected were only a fixed decision for the CNN + HOG + LBP + mRMR + RF pipeline and not the best feature dimension. The architecture is summarised in Figure 7.

The final benchmark implementation therefore separates representation extraction, feature selection, and decision-stage classification into different stages. In the main benchmark, we consider this setup as a complete hybrid CNN + HOG + LBP + mRMR + RF pipeline. The second ablation in Section 4.2 is to investigate the effect of adding handcrafted descriptors to the network while keeping mRMR feature selection and the Random Forest classifier fixed. Note that the feature selection or classifier choice should not be separated from each other. This staged design is one reason the framework is informative in a comparative benchmark: it reduces the risk that all discriminative capacity is tied to a single high-capacity image encoder and instead tests whether structurally motivated handcrafted cues remain useful under cross-dataset transfer.

#### 3.4.2. Framework 2: Transfer-Learning and Semi-Supervised VGG16 Family

The second methodological family contains four related variants that progressively add semi-supervised and representation-learning elements to a common VGG-16 transfer-learning backbone, following the design of Alghamdi et al. [10]:

- TCNN-VGG16: A fine-tuned VGG-16 transfer CNN providing the supervised baseline for this family.
- SSCNN-Pseudo: Extends TCNN with an iterative pseudo-labelling stage that assigns labels to high-confidence unlabelled samples and incorporates them into subsequent training rounds.
- DAE + TCNN-VGG16: Augments TCNN with an unsupervised convolutional denoising autoencoder (DAE) pre-training stage to improve the initial representation before fine-tuning.
- SSCNN-DAE: Combines both the pseudo-labelling strategy and the DAE pre-training, representing the most expressive variant in the family.

**Figure 8:**
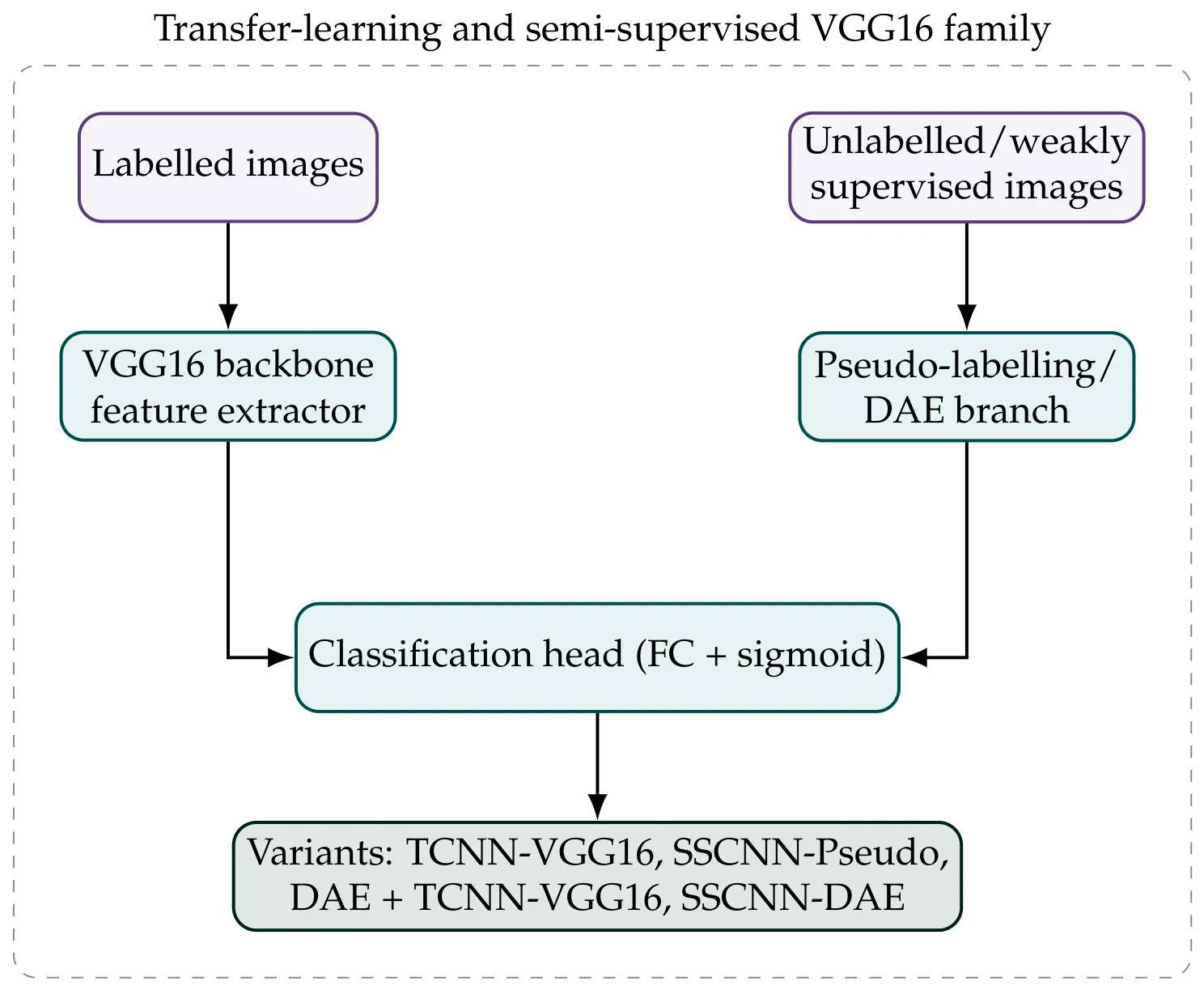
Architecture of Framework 2, comprising the transfer-learning baseline and the three semi-supervised VGG16 variants.

These variants were included to quantify whether semi-supervised design choices, pseudo-labelling, and denoising-based representation learning retain their reported benefits once the evaluation protocol enforces balanced sampling, threshold tuning, repeated-seed averaging, and cross-dataset transfer. The family architecture is shown in Figure 8.

#### 3.4.3. Framework 3: Compact Retinex-Enhanced CNN

**Figure 9:**
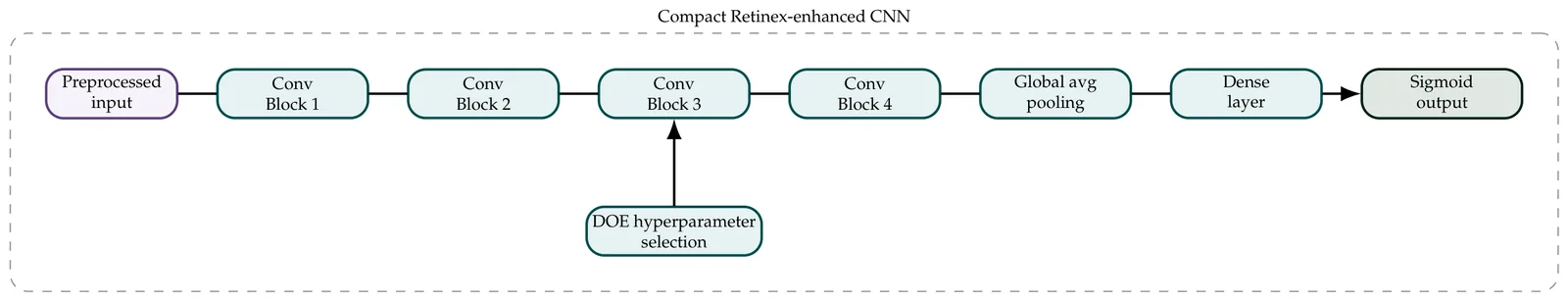
Architecture of the compact Retinex-enhanced CNN with DOE-guided hyperparameter selection (Framework 3).

The third framework is a lightweight four-block convolutional model designed to evaluate whether a substantially smaller network remains competitive when coupled with illumination-aware preprocessing and a structured hyperparameter search. As illustrated in Figure 9, the architecture applies four successive convolutional blocks followed by global average pooling and a single dense layer. Hyperparameter selection was performed using a Design of Experiments (DOE) approach inspired by Song et al. [15], which provides a principled and computationally efficient alternative to exhaustive grid search. This framework provides an important comparison point for computationally constrained deployment scenarios where both model size and inference latency are practical constraints.

The compact CNN was implemented as a four-block convolutional network followed by global average pooling and a single sigmoid classification head. In the benchmark, this architecture was intentionally kept lightweight so that its behaviour could be interpreted as a deployment-oriented baseline rather than as a reduced version of a large transfer-learning model. Hyperparameter selection was guided by a structured DOE procedure rather than unrestricted search, thereby preserving comparability with the other frameworks while limiting over-optimisation on any single dataset. In its strongest within-dataset configuration, the compact model operated with approximately 0.39 million parameters and mean inference latency of 12.84 ms per image, making it substantially smaller than the VGG16-based family while still competitive enough to serve as a meaningful reference for efficiency-sensitive screening scenarios.

### 3.5. Hyperparameter Optimisation and Training Configuration

All models were trained under a unified policy designed to provide fair comparison rather than unconstrained per-model optimisation. Table 4 summarises the key training configuration shared across frameworks. Individual frameworks may use a subset of these settings, as indicated.

For deep-learning models, the training objective was the weighted binary cross-entropy loss:

(4) $$L_{\mathrm{WBCE}}=-\frac{1}{N}\sum_{i=1}^{N}\;\left[w_{1}y_{i}\log{\hat{p}}_{i}+w_{0}\left(1-y_{i}\right)\log\left(1-{\hat{p}}_{i}\right)\right],$$

where $y_{i}\in \left\{0,1\right\}$ is the ground-truth label, ${\hat{p}}_{i}$ is the predicted glaucoma probability, and $w_{0}$, $w_{1}$ are class weights derived from the inverse class frequency of the training split. This weighting scheme penalises errors on the minority class more heavily, mitigating the effect of class imbalance without requiring equal numbers of samples per class.

### 3.6. Training and Evaluation Protocol

All experiments were repeated independently for seeds 0–4. Within-dataset evaluation used the stratified partitions described in Section 2.1, yielding four within-dataset tasks:

R→R,D→D,H→H,O→O.

Cross-dataset evaluation trained on one dataset’s full training partition, tuned on its validation partition, and tested directly on the held-out test partition of each of the three remaining datasets, without any target-domain data exposure. Under the four-dataset configuration this produces $|T_{\mathrm{cross}}|=4\times 3=12$ ordered transfer tasks.

Test-time augmentation was applied during all evaluations by averaging predictions over *K* stochastically transformed views:

(5) $${\hat{p}}_{\mathrm{TTA}}\left(x\right)=\frac{1}{K}\sum_{k=1}^{K}\hat{p}\;\left(T_{k}\left(x\right)\right),$$

where $T_{k}\left(\;\cdot \;\right)$ is the *k*-th stochastic transformation. TTA reduces sensitivity to single-image variability and provides a more stable prediction surface, which is especially important for correctly estimating calibration metrics under small test sets.

A validation-based operating threshold was selected independently for each run. The threshold was chosen to maximise the Matthews correlation coefficient (MCC) on the validation set:

(6) $$\tau^{⋆}=\arg\max_{\tau \in [0,1]}\mathrm{MCC}\left(\tau \right),$$

MCC was preferred over accuracy- or F1-based threshold criteria because it accounts simultaneously for all four entries of the confusion matrix and remains informative under class imbalance, making it more appropriate for screening settings where both sensitivity and specificity matter and where class ratios differ substantially across datasets.

### 3.7. Evaluation Metrics

The benchmark reports both threshold-dependent and threshold-free metrics. Let TP, TN, FP, and FN denote the entries of the binary confusion matrix, where TP represents glaucomatous eyes correctly predicted as glaucoma, TN represents normal eyes correctly predicted as normal, FP represents normal eyes incorrectly predicted as glaucoma, and FN represents glaucomatous eyes incorrectly predicted as normal. The principal threshold-dependent metrics are:

(7) $$\begin{array}{cc} \mathrm{Accuracy} & =\frac{TP+TN}{TP+TN+FP+FN}, \end{array}$$

(8) $$\begin{array}{cc} \mathrm{Sensitivity} & =\frac{TP}{TP+FN}, \end{array}$$

(9) $$\begin{array}{cc} \mathrm{Specificity} & =\frac{TN}{TN+FP}, \end{array}$$

(10) $$\begin{array}{cc} \mathrm{Precision} & =\frac{TP}{TP+FP}, \end{array}$$

(11) $$\begin{array}{cc} F1 & =\frac{2\;\cdot \;\mathrm{Precision}\;\cdot \;\mathrm{Sensitivity}}{\mathrm{Precision}+\mathrm{Sensitivity}}, \end{array}$$

(12) $$\begin{array}{cc} \mathrm{Balanced}\;\mathrm{Accuracy} & =\frac{\mathrm{Sensitivity}+\mathrm{Specificity}}{2}, \end{array}$$

(13) $$\begin{array}{cc} \mathrm{MCC} & =\frac{TP\;\cdot \;TN-FP\;\cdot \;FN}{\sqrt{\left(TP+FP)(TP+FN)(TN+FP)(TN+FN\right)}}. \end{array}$$

The threshold-free discrimination metric is the area under the receiver operating characteristic curve (AUC). Calibration quality is assessed using the Brier score,

(14) $$\mathrm{Brier}=\frac{1}{N}\sum_{i=1}^{N}{\left({\hat{p}}_{i}-y_{i}\right)}^{2},$$

and the expected calibration error (ECE),

(15) $$\mathrm{ECE}=\sum_{m=1}^{M}\frac{|B_{m}|}{N}\left|\mathrm{acc}\left(B_{m}\right)-\mathrm{conf}\left(B_{m}\right)\right|,$$

where $B_{m}$ is the set of predictions assigned to confidence bin *m*, $\mathrm{acc}(B_{m})$ is the empirical accuracy in that bin, and $\mathrm{conf}(B_{m})$ is the mean predicted probability. Low ECE and low Brier score jointly indicate that a model’s probability outputs are trustworthy as clinical inputs to referral or triage decisions, not merely that its binary predictions are correct on average.

### 3.8. Statistical Significance Testing

Performance was compared across models using non-parametric procedures appropriate for repeated-measures experimental designs without distributional assumptions. A Friedman omnibus test was first applied across all six models for each primary metric (accuracy, AUC, balanced accuracy, and MCC), using the per-task, per-seed result as the observation unit. When the Friedman null hypothesis was rejected, pairwise Wilcoxon signed-rank tests with Holm–Bonferroni correction were performed as post-hoc comparisons. This procedure controls the family-wise error rate while remaining more powerful than Bonferroni correction alone. Reporting both the omnibus test and the corrected pairwise comparisons ensures that model-ranking differences are supported by statistically rigorous evidence rather than descriptive observation, which is particularly important in the four-dataset setting where domain shift can otherwise produce unstable rankings that vary with the specific evaluation task [16].

### 3.9. Computational Cost Analysis

In addition to classification performance, the study systematically records parameter count, training time per epoch, total training duration, and inference latency per image. This analysis reflects the practical reality that glaucoma-screening systems are evaluated not only by their predictive accuracy but also by the feasibility of deployment under memory, latency, and infrastructure constraints. The performance–efficiency frontier is of particular clinical relevance: a model with marginally lower AUC but an order of magnitude faster inference and a fraction of the memory footprint may be preferable for large-scale screening programs, especially in resource-limited ophthalmic settings.

### 3.10. Reproducibility and Implementation Details

The entire benchmark was organised as a fully scripted, reproducible pipeline. All steps dataset discovery, label harmonisation, preprocessing, stratified splitting, model training, threshold selection, TTA evaluation, calibration analysis, statistical testing, and figure generation are automated and parameterised so that any experimental outcome can be traced to a specific combination of seed, dataset, model, and preprocessing configuration. The pipeline accepts any subset of the four datasets and automatically determines the applicable within-dataset and cross-dataset tasks. Run-level metrics, seed-aggregated summaries, calibration statistics, Friedman-test results, and Wilcoxon post-hoc comparisons are all recorded at experiment time and form the basis of all reported results.

## 4. Results

In this section, we report the objective results of our four-dataset glaucoma-screening benchmark on RIM-ONE, DRISHTI-GS, HYGD, and ORIGA. We evaluate six model configurations across three families of methods in different preprocessing pipelines with repeated runs, calibration analyses, computational efficiency regression, and non-parametric statistical tests. We report the results in terms of within-dataset performance, cross-dataset transfer performance, preprocessing effects, calibration results, threshold variation, computational efficiency, seed variability, and statistical comparisons. The clinical value, deployment readiness, threshold portability, site-specific retuning, and recommendations for external validation are left for discussion in Section 5.

**Figure 10:**
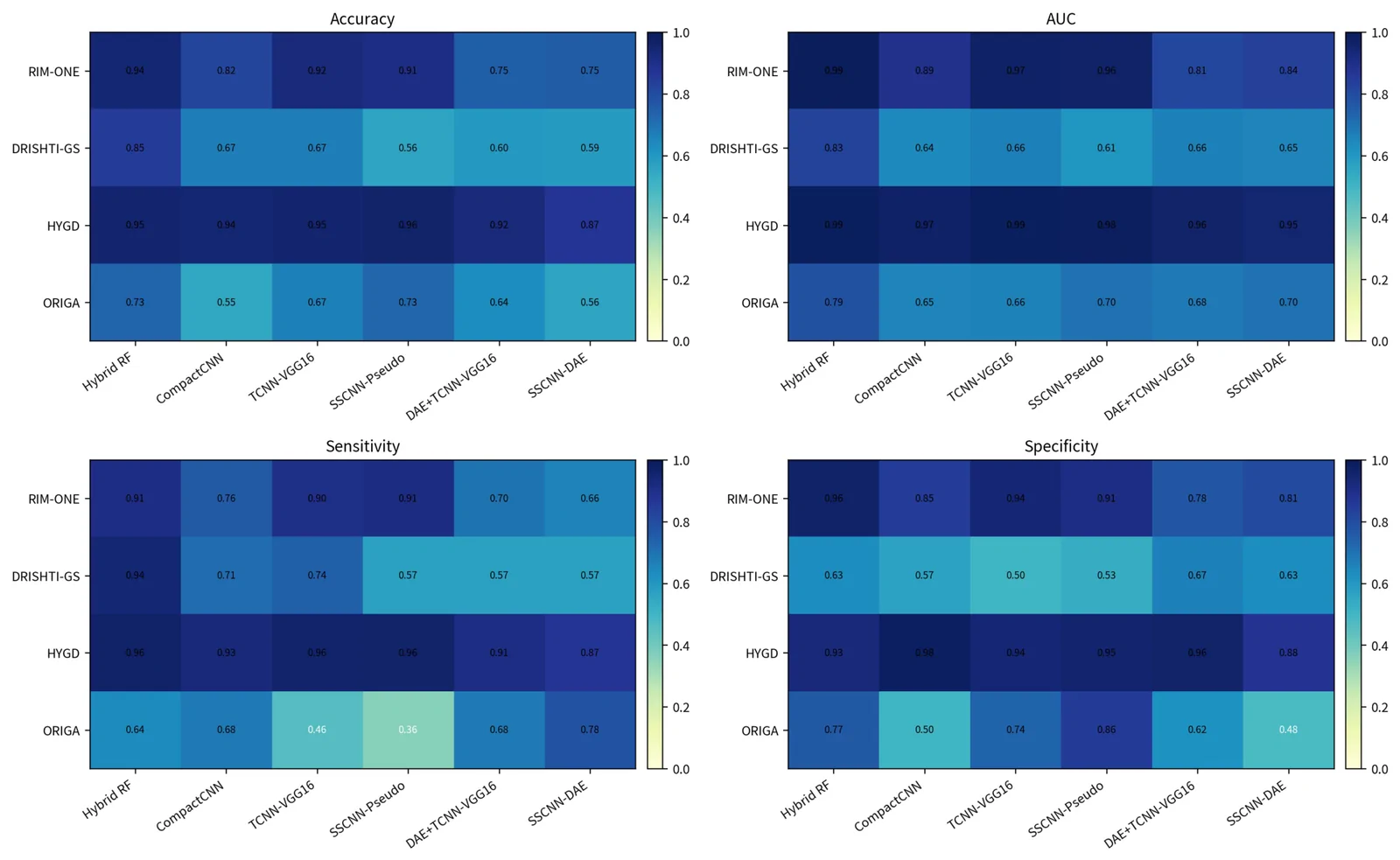
Mean model performance when trained and tested on the same dataset. The hybrid random forest achieves the highest accuracy and area under the curve.

**Figure 11:**
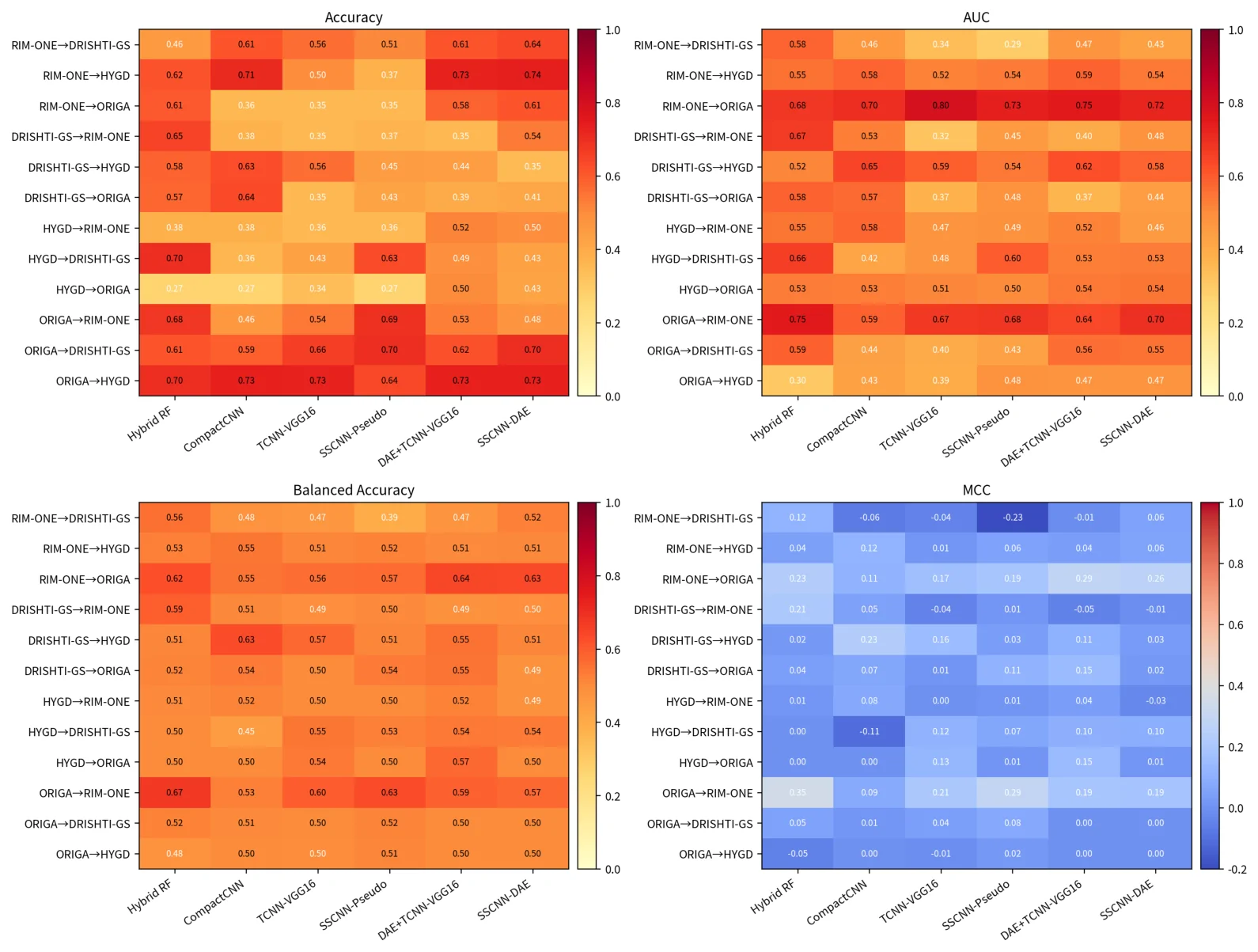
Cross-dataset heatmaps (best MCC configuration). Accuracy, AUC, balanced accuracy, and MCC for models trained on one dataset and tested on another, showing reduced and variable performance under distribution shift.

**Figure 12:**
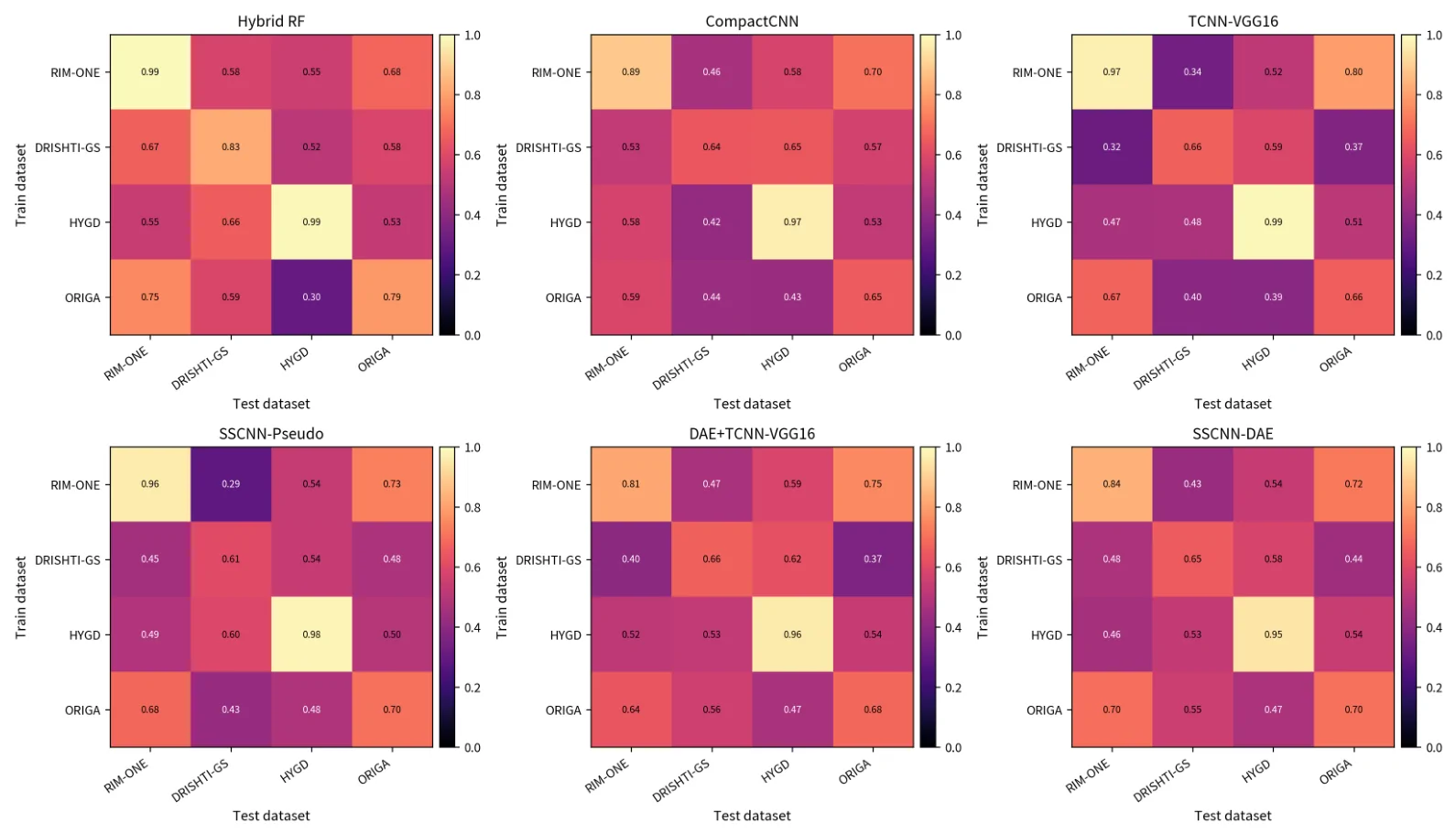
AUC transfer matrices by model. Train–test AUC values across RIM-ONE, DRISHTI-GS, HYGD, and ORIGA. Diagonal entries indicate within-dataset performance, while off-diagonal values reflect cross-dataset generalisation, demonstrating reduced and variable transferability across domains.

**Figure 13:**
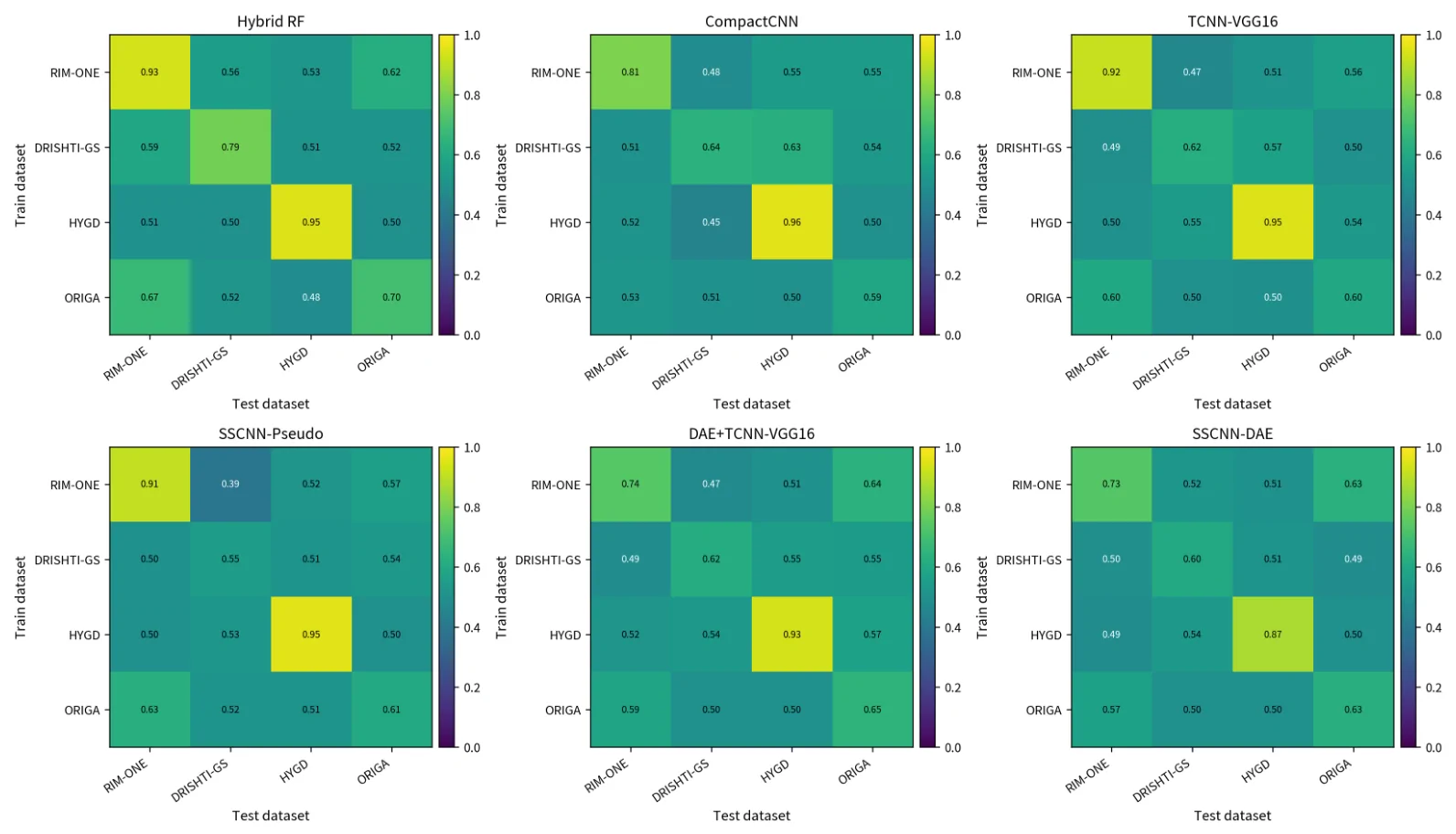
Train–test balanced accuracy across RIM-ONE, DRISHTI-GS, HYGD, and ORIGA. Diagonal values show within-dataset performance; off-diagonal values reflect cross-dataset generalisation with moderate but reduced transfer stability.

**Figure 14:**
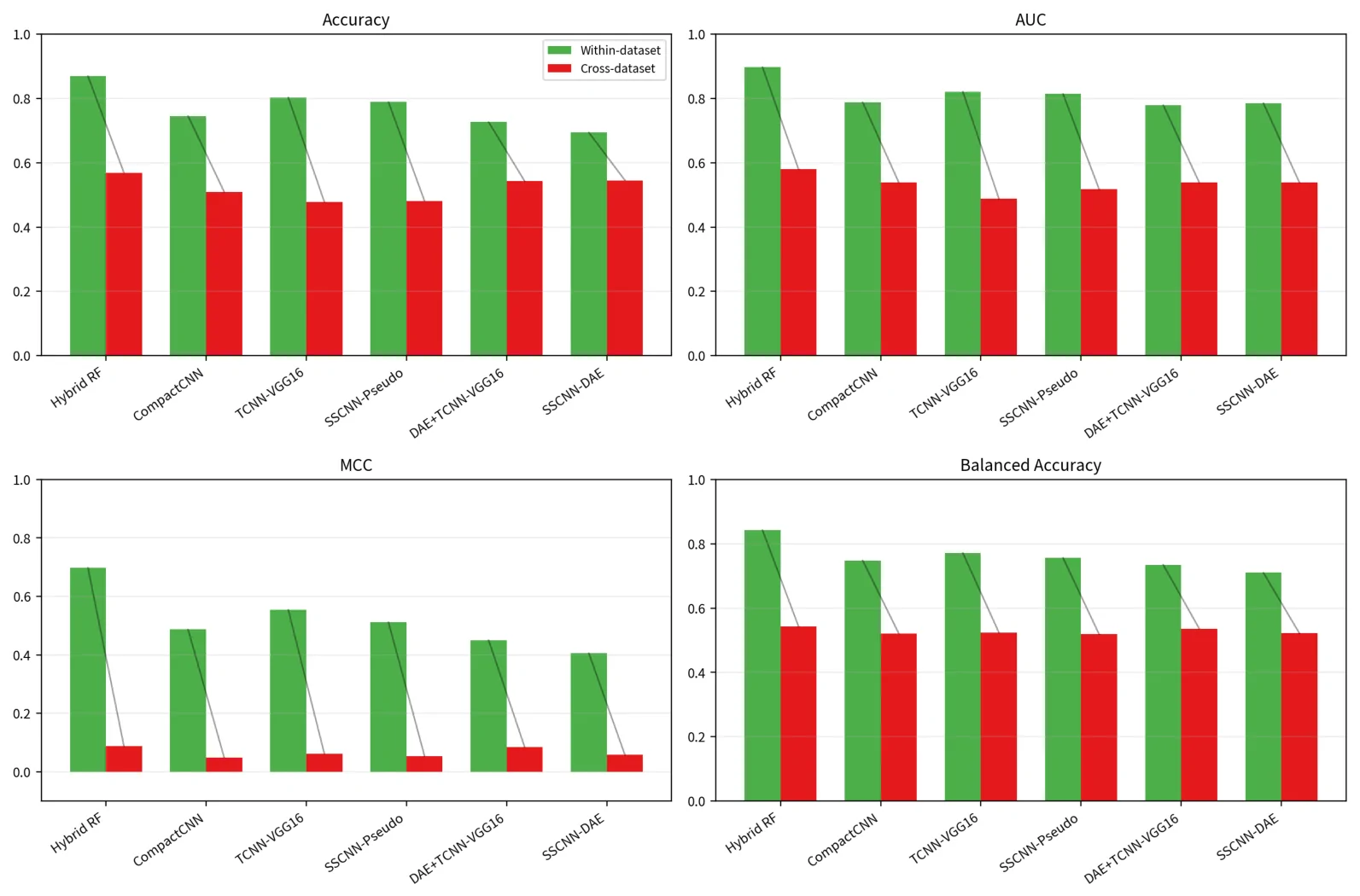
Mean Accuracy, AUC, MCC, and Balanced Accuracy for each model under matched (within) and external (cross) evaluation. All models show substantial performance decline under cross-dataset transfer, particularly in MCC.

**Figure 15:**
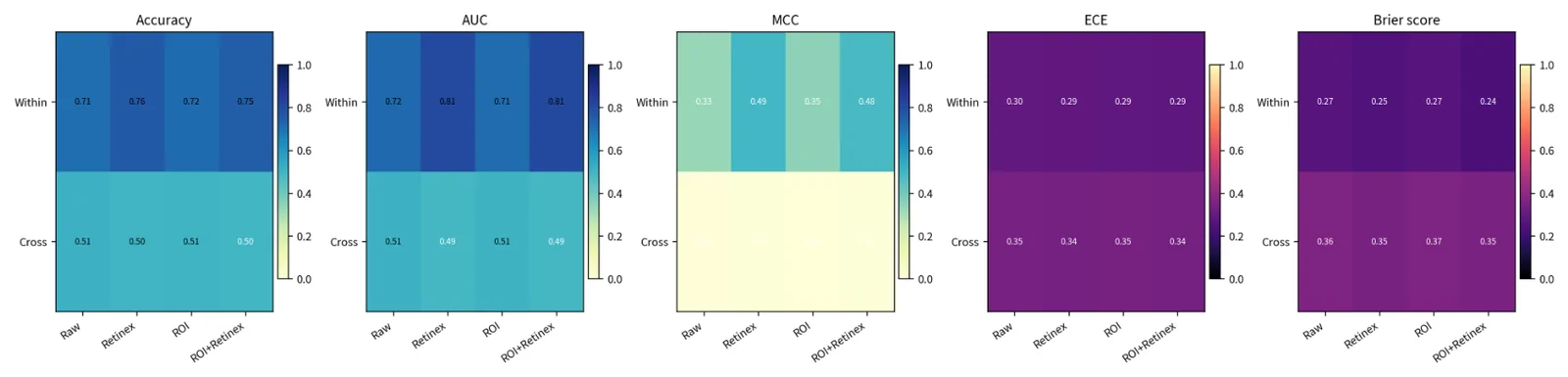
Effect of preprocessing on discrimination and transportability. Raw images provided the best mean within-dataset AUC, whereas ROI preprocessing yielded the strongest cross-dataset accuracy and Retinex preprocessing improved cross-dataset MCC. This pattern suggests that preprocessing may alter not only image quality but also the domain dependence of learned features. In clinical AI development, preprocessing should therefore be justified by its effect on external validity rather than internal performance alone.

**Figure 16:**
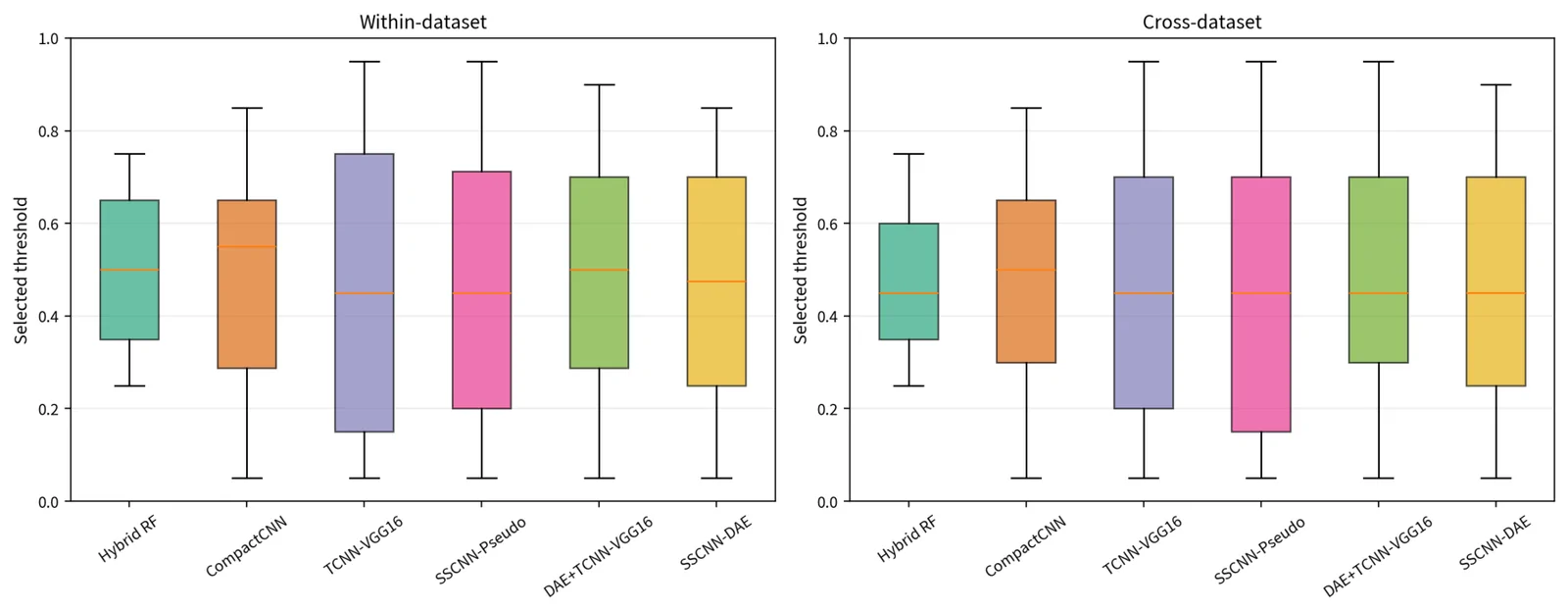
Drift in selected operating thresholds across domains. Large variation indicates limited portability of decision rules and the need for site-specific tuning, which complicates consistent sensitivity and specificity targets in glaucoma screening.

**Figure 17:**
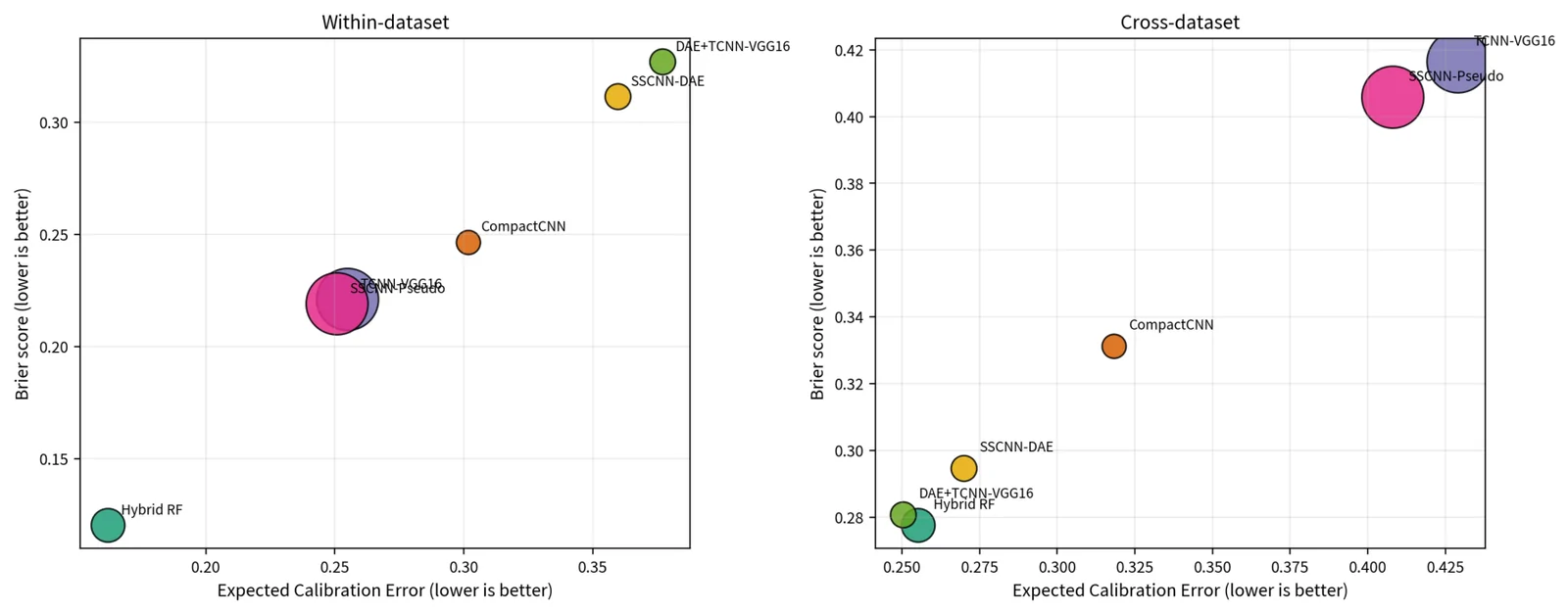
Calibration-quality trade-off across models.

**Figure 18:**
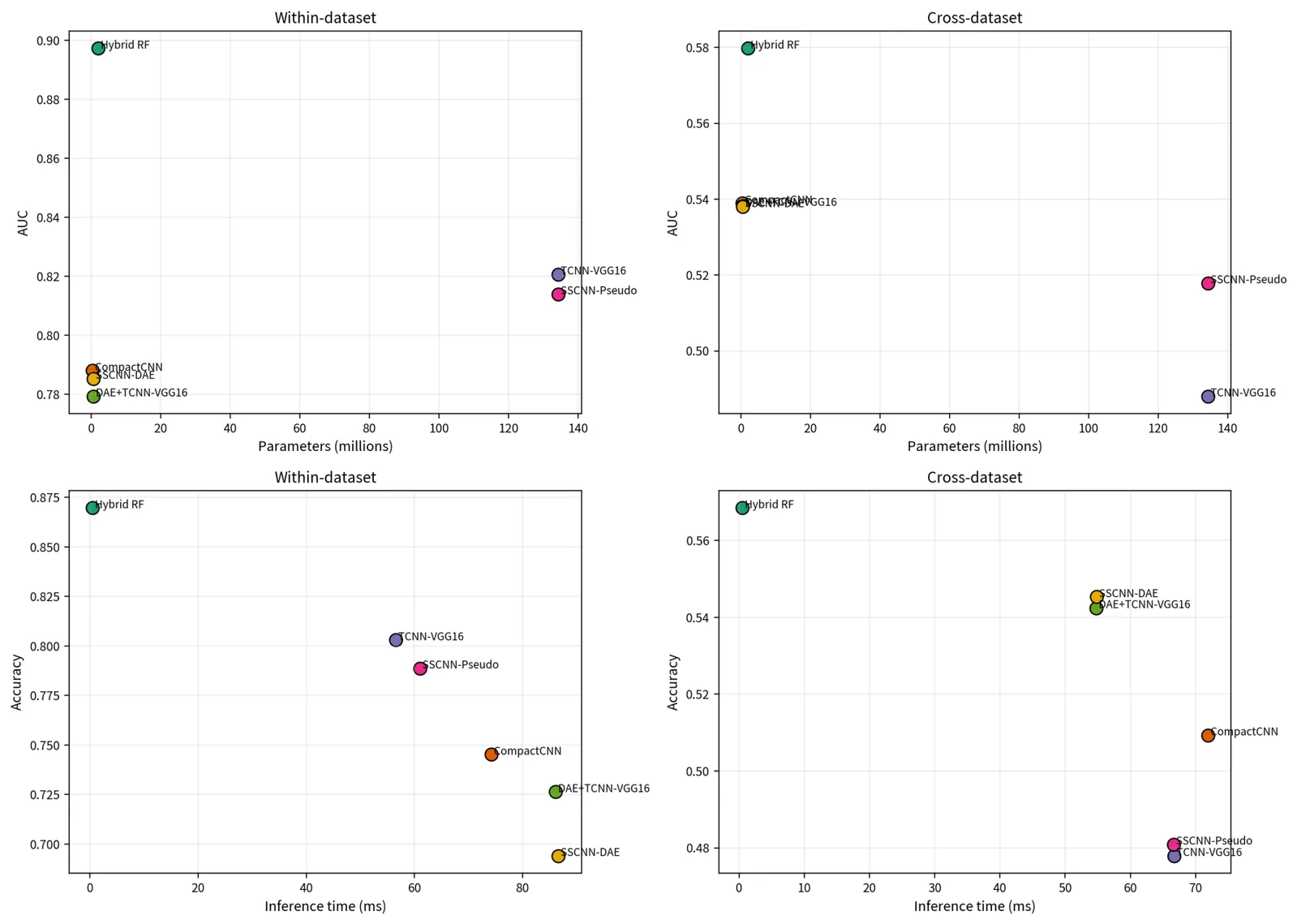
Efficiency frontier of predictive performance vs computational cost.

**Figure 19:**
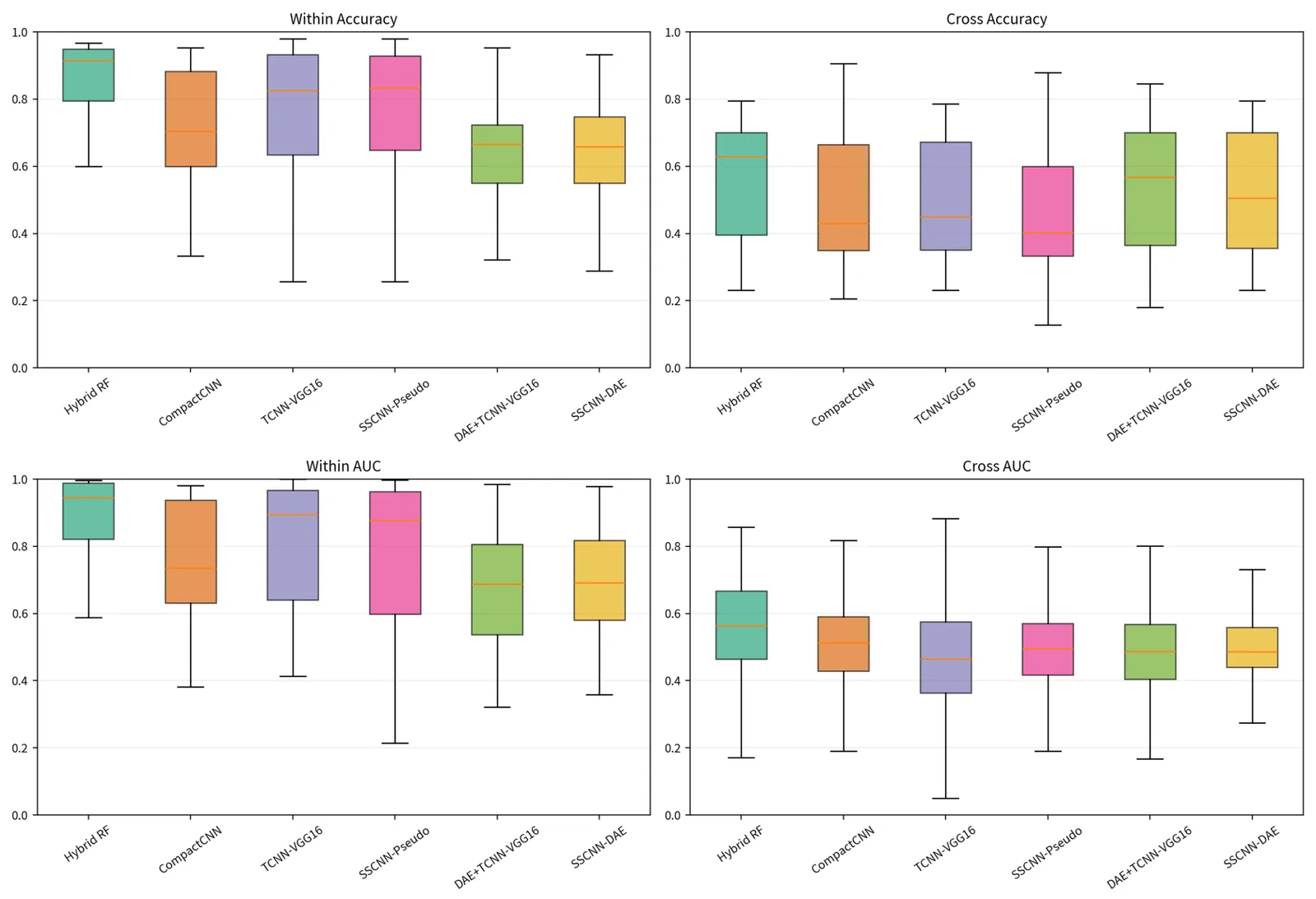
Seed-level performance distributions. Boxplots show run-to-run variability across datasets, models, and preprocessing settings.

**Figure 20:**
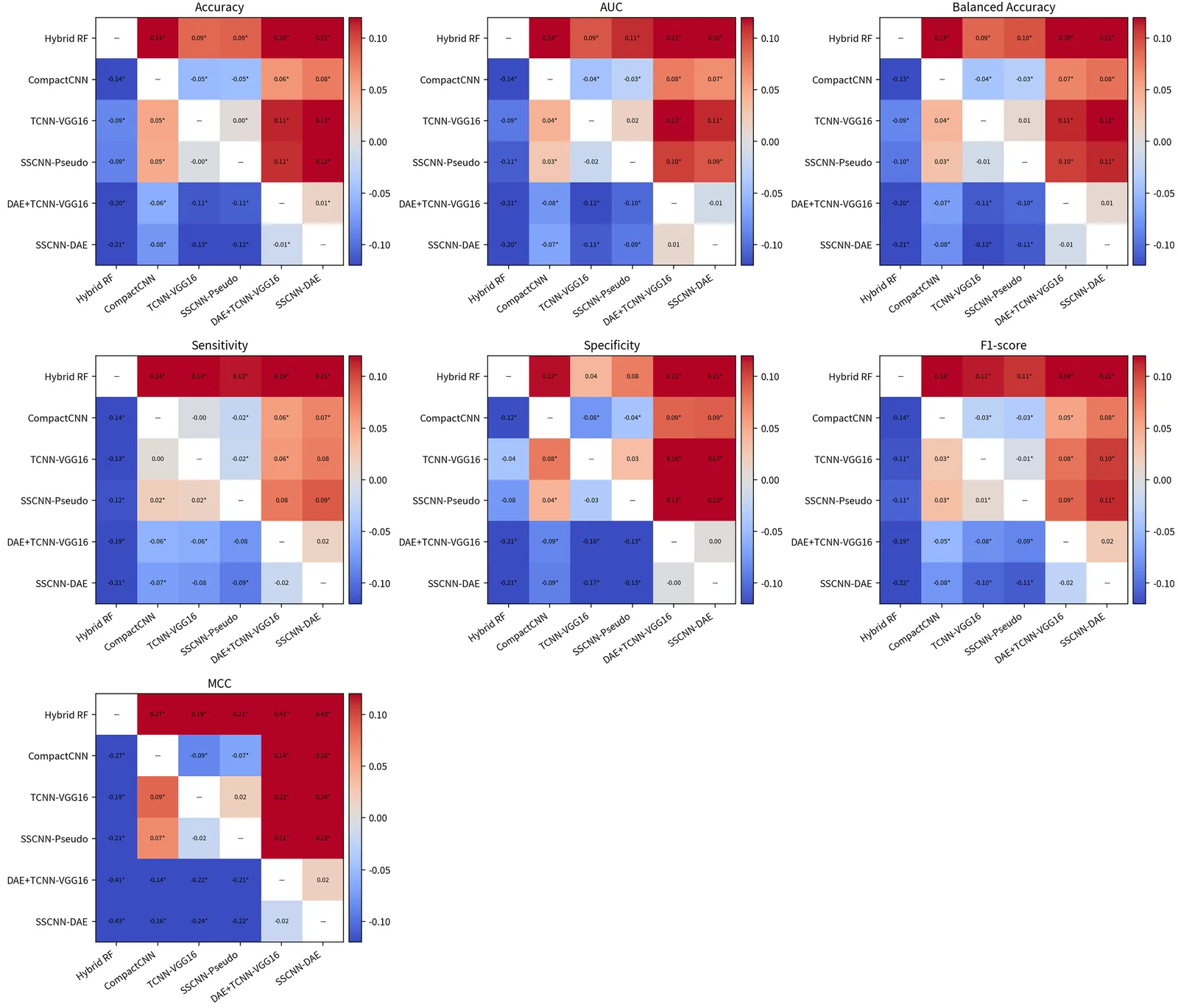
Wilcoxon signed-rank significance heatmaps for pairwise model comparisons. Heatmaps display adjusted *p*-values and effect directions across evaluation metrics. * indicates statistically significant pairwise differences after correction for multiple comparisons.

**Figure 21:**
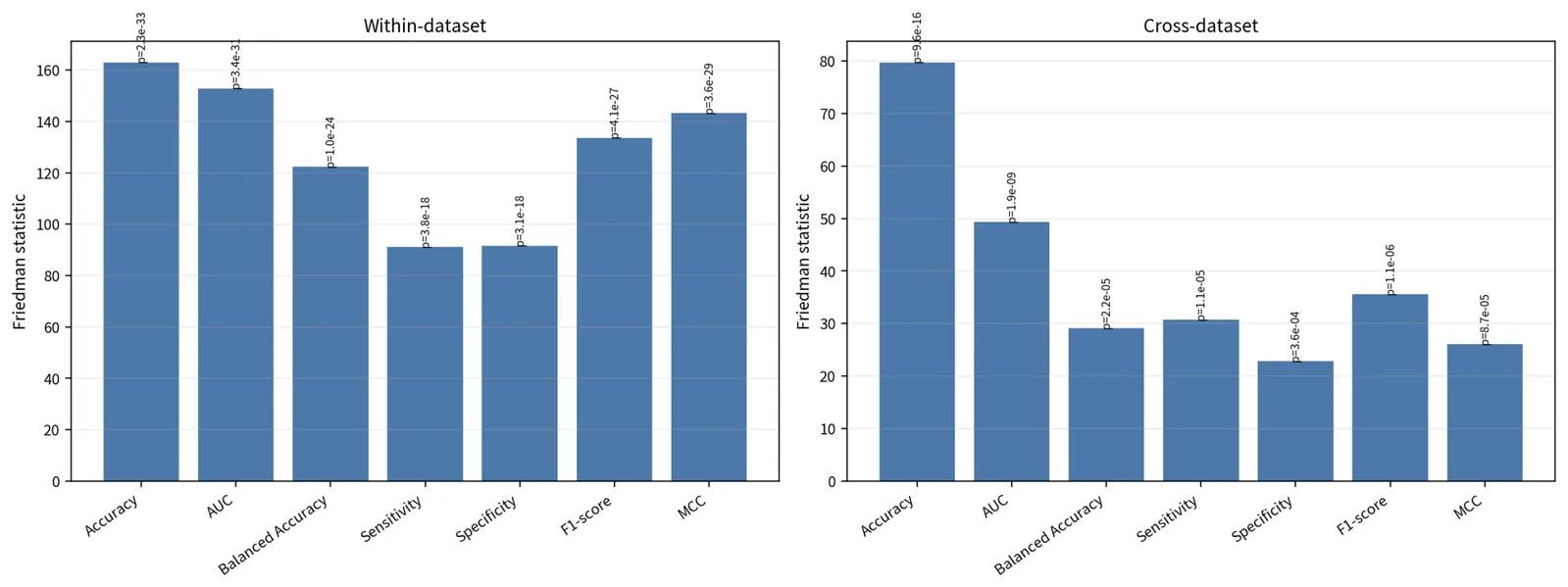
Friedman omnibus test summary across key metrics. Significant results across all principal metrics indicate systematic differences among models rather than chance variation, supporting consistent ranking patterns in glaucoma-screening evaluation.

**Figure 22:**
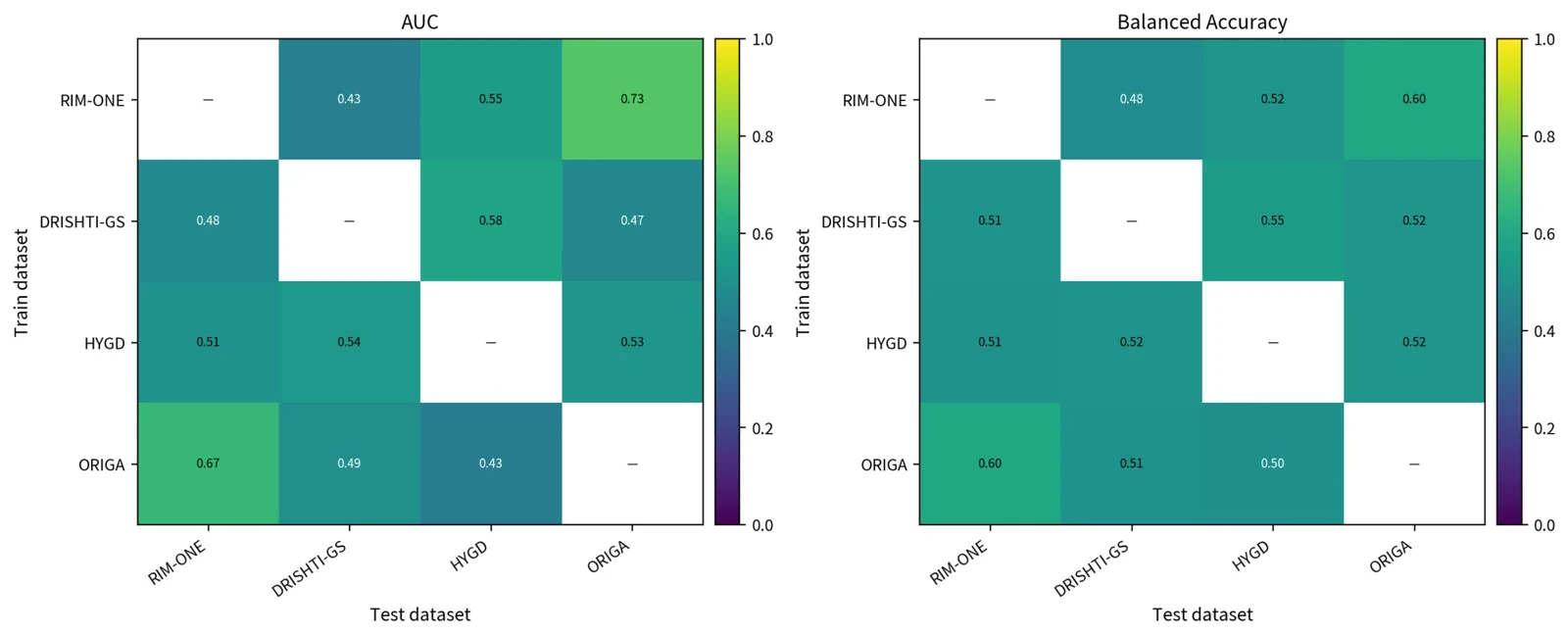
Cross-dataset difficulty map. Aggregated performance across models highlights intrinsically challenging source–target pairs.

**Figure 23:**
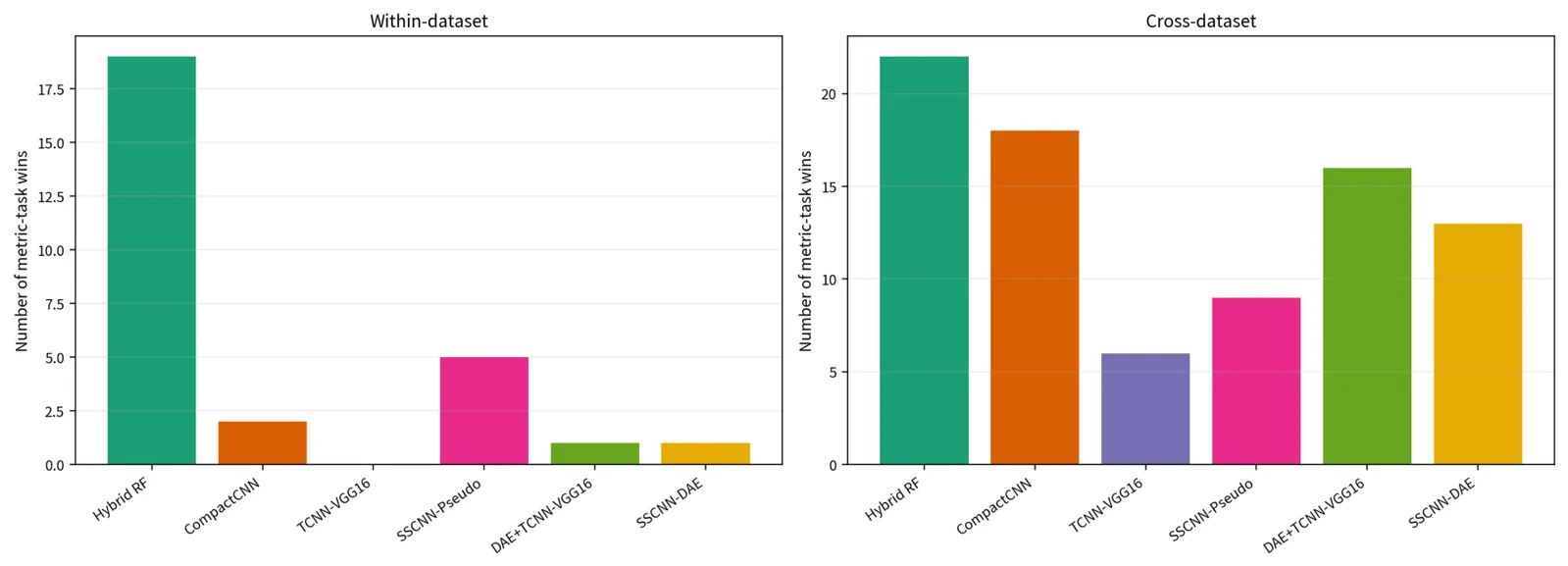
Winner-count summary across tasks and metrics. Counts of top-performing results per model across the benchmark.

For interpretive clarity, the results progress from internal to deployment-oriented evidence. We first report within-dataset performance, which estimates model behaviour when training and testing occur on the same source. We then examine cross-dataset transfer, which provides the more stringent test of external validity. The remaining subsections analyse preprocessing effects, calibration quality, threshold stability, computational efficiency, seed-level variability, and statistical significance. Taken together, Figure 10, Figure 11, Figure 12, Figure 13, Figure 14, Figure 15, Figure 16, Figure 17, Figure 18, Figure 19, Figure 20, Figure 21, Figure 22 and Figure 23 provide a descriptive summary of internal discrimination, cross-dataset transfer, calibration behaviour, threshold variation, computational efficiency, seed-level variability, and statistical ranking. The clinical and deployment-oriented interpretation of these findings is provided in Section 5.

### 4.1. Overall Within-Dataset Performance

In the context of within-dataset evaluation, it is the most robust test to assess how bad cross-dataset degradation is. When training and testing are performed on the same dataset, the performance of the models differs considerably across methodological families, as shown in Figure 10. As shown in Figure 10, Hybrid RF achieved the highest performance in within-dataset discrimination, achieving the highest mean accuracy (0.8697), AUC (0.8972), MCC (0.6983) and balanced accuracy (0.8428). Moreover, it also has the lowest mean ECE (0.1621) and Brier score (0.1202) and the best calibration quality. TCNN-VGG16 and SSCNN-Pseudo are second in performance while CompactCNN and SSCNN-DAE are less competitive when we compare them from different sources.

While Figure 10 provides a good perspective of matched-source performance, Table 5 summarizes the main benchmark findings in a concise manner. Table 6 and Table 7 further show that the best-performing within-dataset configuration and the best-performing model varied by dataset and preprocessing pipeline, indicating that internal superiority was not uniform across model families or cohorts. Table 5 shows that Hybrid RF was not only the strongest matched-source model but also the most robust discriminator under cross-dataset comparison. We note that while DAE + TCNN-VGG16 is not the strongest in discrimination, it provides a better probability calibration under cross-dataset comparisons. This indicates that the model with the strongest discrimination was not necessarily the model with the lowest calibration error under cross-dataset evaluation; the implications of this distinction are discussed in Section 5.

Table 6 complements this benchmark-level summary by reporting the strongest within-dataset configuration observed for each model family. This model-level view shows that the best-performing internal configurations were not uniformly associated with the same preprocessing pipeline, reinforcing that performance depended not only on architectural design but also on the representation presented to the classifier. In addition, Table 7 provides the strongest matched-source result for each dataset individually. This dataset-level view is important because it shows that the leading internal performer was not identical across all four datasets, indicating that internal superiority remained partly source dependent. From a clinical imaging perspective, this is unsurprising: the four datasets differ in class balance, image appearance, and likely acquisition conditions, so a model that dominates one cohort cannot automatically be assumed to dominate another.

### 4.2. Feature-Representation Ablation of the Hybrid Feature-Fusion Framework

To examine the contribution of feature representation in the hybrid deep-handcrafted framework, we included an additional feature-representation ablation. The mRMR feature-selection and Random Forest classifier were kept fixed and the input feature set was varied by four progressively enriched configurations: (i) deep CNN embedding alone, (ii) CNN combined with HOG features, (iii) CNN combined with LBP descriptors and (iv) the full CNN + HOG + LBP configuration used throughout the main benchmark. In the latter two configurations, the effect of handcrafted descriptors can be evaluated only in a fixed downstream selection and classification pipeline. However, this is not a full factorial ablation of the complete hybrid model, since the independent effects of mRMR feature selection and Random Forest classifier are not separately evaluated. Each configuration was processed in the same 70:10:20 protocol and repeated five times for each of the four datasets, for both within-dataset and across-dataset.

Table 8 presents the mean within-dataset and cross-dataset accuracy and AUC obtained for each feature configuration. The results show that the deep CNN embedding alone provides a reasonable within-dataset baseline, and that adding HOG and LBP descriptors improves both within-dataset discrimination and cross-dataset transportability when mRMR and the Random Forest classifier are held fixed. The full CNN + HOG + LBP configuration, corresponding to the Hybrid RF model reported throughout the remainder of this study, achieved the strongest performance among the four tested feature configurations.

These findings support the idea that handcrafted gradient and texture-based descriptors provide complementary information to the CNN embedding under the fixed mRMR+RF downstream pipeline. However, this ablation should not be interpreted as evidence that feature fusion alone is responsible for the whole Hybrid RF advantage as the independent contribution of mRMR feature selection and Random Forest classification was not evaluated separately.

### 4.3. Cross-Dataset Generalisation and Domain Shift

Cross-dataset evaluation is the central test of model transportability, as it considers whether a detector trained in one source could still be useful in an unseen external population. As can be seen in Figure 11, all models in this investigation perform decently and the good within-dataset results do not translate to good external behaviour. The cross-dataset accuracy, AUC, MCC, and balanced accuracy all drop dramatically compared to matched-source evaluation, indicating that the performance of the data shifted considerably.

Despite this overall erosion, Hybrid RF was also the best model to transfer and achieved the highest mean cross-dataset accuracy of 0.5685, AUC (0.5798), MCC (0.0871) and balanced accuracy of 0.5429. However, these values were still significantly below its within-dataset performance and show that it is difficult to maintain diagnostic utility across institutions and acquisition conditions. DAE + TCNN-VGG16 was close behind, with the transfer accuracy of 0.5424 and relatively good calibration behaviour. TCNN-VGG16 and SSCNN-Pseudo both showed a larger within-to-cross performance decline and showed more sensitivity to source-specific image features and a higher degree of domain dependence.

The transfer matrices in Figure 12 and Figure 13 show a more detailed picture of this gap in generalisation. In both matrices, the diagonal entries indicate that a system is being evaluated for the matched source and off-diagonal data to the external source that is being transferred. Because of the close comparison between the results in the diagonal and off-diagonal cases, the researcher showed that the particular characteristics of the dataset played a very important role in both ranking-based and threshold-dependent work. This is clinically relevant because the glaucoma screening systems are expected to be able to do work in a wide range of acquisition scenarios and not just under the limited test conditions.

The within-versus-cross comparison in Figure 14 is especially clear. The within-to-cross drop is a good rough estimate of domain sensitivity. TCNN-VGG16 exhibited the largest drop and is still relatively weakly resistant to external changes despite its strong matched-source performance. Overall, we find that we could have expected internal validation to have given a much higher value for the quality of our models than we can expect, even if our models are in realistic deployment conditions.

Table 9 complements the heatmaps and transfer matrices by reporting the best-performing configuration for each ordered source-to-target transfer pair. This table is particularly informative because it makes the asymmetry of cross-dataset transport explicit: some transfer directions were substantially more difficult than others, and the best model-preprocessing combination was not constant across source-target pairs. External validity in glaucoma screening is therefore shaped not only by model family, but also by the interaction between source cohort, target cohort, and preprocessing strategy.

To make the degradation from matched-source to external evaluation numerically explicit, Table 10 reports the mean within-dataset and cross-dataset AUC and balanced accuracy for each model together with the corresponding generalisation gap. The table shows that the strongest internally performing models also experienced substantial transfer losses, reinforcing that internal superiority does not imply robust external validity.

Table 10 confirms that every model family exhibited a substantial loss of performance under transfer. Importantly, the strongest internally performing models did not necessarily experience the smallest degradation. Hybrid RF remained the strongest external discriminator not because its relative loss was smallest, but because it started from the strongest matched-source baseline and therefore retained the highest absolute transfer performance. This result reinforces the need to evaluate retained external performance and generalisation gap together rather than in isolation.

Overall, the modest absolute transfer results across all model families show that cross-dataset performance remained limited under the present zero-shot evaluation protocol. The deployment implications of this finding are discussed in Section 5.

### 4.4. Influence of Preprocessing on Transportability

Preprocessing had a measurable but non-uniform effect on performance. As shown in Figure 15, raw images yielded the best mean within-dataset AUC, indicating that aggressive transformation was not necessary to maximise internal discrimination. By contrast, ROI-based preprocessing yielded the strongest cross-dataset accuracy, whereas Retinex preprocessing was associated with the strongest cross-dataset MCC. These findings indicate that preprocessing should not be regarded as a universally beneficial enhancement; rather, its value depends on whether the objective is to maximise matched-source discrimination or to improve external transportability.

This distinction is important in glaucoma screening because preprocessing can subtly alter the extent to which a model relies on site-specific appearance cues. A pipeline that improves performance on one dataset may reduce portability if it amplifies dataset-specific cropping conventions, illumination characteristics, or contrast patterns. The four-dataset evaluation therefore provides a more realistic basis for selecting preprocessing strategies than an analysis confined to a single source.

### 4.5. Calibration Quality and Operating-Point Stability

Calibration quality was evaluated in addition to discrimination because probability reliability is a distinct model property. Under cross-dataset evaluation, DAE + TCNN-VGG16 achieved the lowest mean ECE (0.2504), indicating the most reliable predicted probabilities under transfer among the evaluated models. Hybrid RF remained competitive on calibration while preserving the strongest overall discrimination, but DAE + TCNN-VGG16 occupied a particularly favourable region of the calibration–discrimination trade-off space. This shows that discrimination and calibration ranked the models differently under transfer.

Threshold stability provides a complementary perspective on deployment suitability. Figure 16 visualises the drift in selected operating thresholds across datasets and runs. Large threshold variation indicates that a model’s decision rule is not portable and may require site-specific re-tuning to preserve a predictable sensitivity–specificity balance. In practice, such instability is undesirable because it complicates prospective calibration and weakens confidence that a threshold selected in one environment will remain appropriate in another. Calibration quality and threshold stability should therefore be considered together when prioritising models for external validation.

Figure 17 shows the discrimination and calibration quality by plotting the expected calibration error (ECE) against Brier score in both the within-dataset and cross-dataset settings. Each point represents the average performance of a model in each task and allows for comparison of probability reliability beyond binary accuracy. Within-dataset, Hybrid RF falls in the lower left of the plot, indicating both low ECE and low Brier score, which is consistent with its good matched-source calibration. When we transfer data to cross-dataset, the calibration degradation is clear for all models. Most points are higher in the ECE and Brier, and DAE + TCNN-VGG16 is in the lower calibration error region than the other deep models. Our analysis in this two-dimensional calibration space indicates that discrimination performance is not the only determinant of clinical utility but that models differ significantly in how well their predicted probabilities reflect actual outcomes after changing domains. This is particularly true in screening applications where referral thresholds are probability-based (i.e., not binary) rather than fixed binary outputs.

Table 11 summarises the mean selected threshold, Brier score, and expected calibration error for each model under both within-dataset and cross-dataset evaluation. Two clinically relevant patterns emerge. First, Hybrid RF showed the most favourable overall calibration profile under matched-source testing. Second, DAE + TCNN-VGG16 remained comparatively strong under external transfer, indicating that its predicted probabilities were more reliable than those of several competing models even when its raw discrimination was not uniformly dominant. These findings support the argument that probability reliability and threshold portability should be assessed alongside AUC and accuracy in glaucoma-screening benchmarks.

To complement the calibration analysis with a more deployment-oriented view of operating-point behaviour, Table 12 reports the mean selected decision threshold under within-dataset and cross-dataset evaluation together with the absolute threshold shift. Lower threshold shift suggests greater operating-point portability across domains, although this should be interpreted jointly with calibration quality rather than in isolation. It shows that threshold shift alone is not sufficient to characterize deployment readiness. Some models exhibited small mean threshold shifts but remained poorly calibrated under external transfer, whereas DAE + TCNN-VGG16 combined a small threshold shift with comparatively favourable cross-dataset calibration. This reinforces the broader conclusion that operating-point stability and probability reliability should be assessed together when selecting models for screening deployment.

### 4.6. Computational Efficiency and Practical Deployment Considerations

Although discrimination and calibration are central to model assessment, computational efficiency also remains important for screening workflows, particularly in resource-constrained settings. The efficiency frontier in Figure 18 shows that the best-performing models did not all occupy the same region of the cost–performance landscape. Hybrid RF combined strong overall performance with a favourable efficiency profile, whereas several deeper models incurred substantially higher computational burden without proportionate gains in cross-dataset transportability.

These results suggest that parsimonious or hybrid approaches may be especially well suited for scalable glaucoma-screening systems when external robustness is at stake and operational effectiveness is at stake. In other words, the most expensive approach might not be the most interesting architecture in terms of transportability and efficiency when all three are taken into account. Table 13 puts this trade-off in numerical terms by providing a representative number of parameters in each model as well as the training time and inference time in the model. This table shows that Hybrid RF was the best at predicting robustness and in terms of operational efficiency, but further down the spectrum the cost of computation is much higher and external behaviour is not improved. As in glaucoma screening, throughput, hardware and service cost are more important in clinical practice than in other applications.

### 4.7. Variability Across Random Seeds

The seed-level distribution analysis in Figure 19 shows that variability was non-negligible across models and tasks, reinforcing the importance of repeated-run reporting rather than single-split performance claims. Some models that appeared competitive in terms of mean performance displayed broader inter-run dispersion, implying reduced reproducibility. This is an important methodological consideration in medical imaging benchmarks, where optimistic results from a single seed may not survive reruns or external replication.

The seed-level analysis therefore refines the interpretation of mean performance. Models that achieve favourable averages but exhibit high variance are less reliable candidates for translation, because their behaviour may be sensitive to initialisation, sampling noise, or minor differences in split composition. Experimental stability is therefore a relevant performance attribute in its own right.

### 4.8. Statistical Significance of Model Ranking

To determine whether the observed ranking patterns reflected more than random variation, both pairwise Wilcoxon signed-rank testing and omnibus Friedman testing were performed. The Wilcoxon analysis, summarised in Figure 20, revealed several highly significant pairwise differences, including Hybrid RF versus SSCNN-Pseudo for cross-dataset accuracy (Holm-adjusted $p=5.68\times 10^{-14}$; Δ=0.109) and Hybrid RF versus SSCNN-DAE for cross-dataset AUC ($p=1.19\times 10^{-6}$; Δ=0.0629), as well as strong within-dataset advantages for Hybrid RF on major endpoints. These pairwise comparisons support the practical superiority of Hybrid RF on the principal discrimination metrics within this benchmark.

The omnibus Friedman tests, shown in Figure 21, were significant across the principal metrics, confirming that the observed ranking differences across models were systematic rather than incidental.

Table 14 complements the figures by summarising both the omnibus tests and representative Holm-corrected Wilcoxon comparisons. Together, these results strengthen the inferential basis of the manuscript by showing that the reported hierarchy is supported not only visually, but also statistically.

### 4.9. Task Difficulty and Broad Model Dominance

The cross-dataset difficulty map in Figure 22 shows that some source-to-target transfers were consistently difficult for all models, suggesting that performance degradation cannot be attributed solely to algorithm choice. Rather, certain dataset pairs likely encode substantial shifts in phenotype distribution, image acquisition conditions, cropping policy, or annotation practice. This is clinically important because it implies that external validation should stress-test such difficult transfers rather than rely only on favourable or closely matched cohorts.

The winner-count analysis in Figure 23 provides a more comprehensive summary of benchmark leadership across tasks and metrics. Hybrid RF was the most successful in terms of task wins, suggesting that the leadership was not limited to a specific setting. Further, we see in the calibration results that no model dominated in every clinically relevant axis at once and DAE + TCNN-VGG16 was still a good choice when reliable probability estimation was the priority. In summary, the benchmark identifies the top model in terms of overall robustness but also shows the trade-offs that we should consider before deploying it.

## 5. Discussion

The glaucoma-screening models have been evaluated under conditions that are closer to real deployment than in single-dataset testing. By benchmarking six model implementations across four public datasets, within and outside the dataset, we can separate internal discrimination performance into external transportability. There are several conclusions we can draw from this design. The first and most important finding is that strong within-dataset performance failed to translate to strong external performance. Although the strongest models achieved high discrimination when training and testing on the same source, transfer (and thus their performance) significantly decreased for all model families. This is in line with the general medical AI literature, where internal test performance tends to overestimate the potential performance of the model when the development and deployment are different [2,3,8]. In our case, we can see that the difference in the diagonal and off-diagonal entries on transfer matrices is still significant as far as dataset identity is concerned. In the clinical setting, we have a very important finding in that glaucoma-screening algorithms are rarely adopted in the same acquisition setting in which they were developed, and a model that is well performing only on the same source makes very little translation into the real world.

The within-dataset results reported in Section 4.1 confirm that high diagnostic performance can be achieved when training and test images are drawn from the same distribution and that hybrid feature learning remains competitive with more computationally demanding deep architectures under internally matched conditions. However, such favorable within-dataset performance should not be interpreted as evidence of deployment readiness: internally favorable results may be partly attributable to dataset-specific imaging characteristics, such as optic-disc framing, contrast, annotation practice, and disease spectrum, rather than to a generalisable glaucoma signal.

The approximately 30-percentage-point drop in mean accuracy for the best-performing model (0.8697 within-dataset versus 0.5685 cross-dataset for Hybrid RF, Table 5) warrants closer examination. We identify four likely contributing and interacting factors. First, acquisition and device heterogeneity: RIM-ONE, DRISHTI-GS, HYGD, and ORIGA were collected using different camera systems and clinical protocols across different countries, altering the low-level colour, contrast, and illumination statistics on which several models partly rely. Second, prior-probability shift: glaucoma prevalence differs substantially across cohorts, ranging from approximately 25.8% in ORIGA and 35% in RIM-ONE to 69% in DRISHTI-GS and 73.4% in HYGD (Table 3); a classifier whose decision threshold is tuned under one prevalence regime is expected to misclassify systematically under a markedly different disease prior, independent of any true change in discriminative ability. Third, annotation and framing-convention differences: RIM-ONE and ORIGA remain optical-disc-centered after preprocessing, whereas HYGD and DRISHTI-GS retain full-image content (Figure 6), so models trained on cropped, disc-centered imagery may encounter substantially different spatial statistics at test time. Fourth, image-quality heterogeneity: HYGD alone provides explicit per-image quality scores, and comparable metadata is unavailable for the other three datasets, making quality variation a plausible but not directly measurable contributor. Consistent with this multi-factorial explanation, Table 9 shows that transfer difficulty is highly asymmetric across source–target pairs rather than uniform, indicating that no single factor alone accounts for the observed decline. Clinically, these findings imply that a glaucoma-screening model cannot be assumed to generalise safely to a new clinic, camera, or population without prospective, site-specific validation and, where feasible, recalibration of both the decision threshold and the underlying probability estimates.

The second important result is that the complete Hybrid RF pipeline was able to achieve the best overall discrimination, external transportability and computational efficiency among the evaluated configurations. In matched-source study, it was able to provide the best overall discrimination in terms of mean accuracy, AUC, MCC and balanced accuracy and to achieve the best absolute cross-dataset transfer performance. One possible explanation for this is that CNN-derived features, handcrafted gradient and texture descriptors, mRMR feature selection and Random Forest classification could reduce the dependence on source-specific image appearance compared with larger end-to-end networks. This should not be taken as a sign that handcrafted or hybrid models are better than deep networks, nor as a sign that feature fusion alone was the reason for the performance advantage. In this benchmark we found that the complete CNN + HOG + LBP + mRMR + RF configuration provided the best overall discrimination, external transportability, calibration and computational efficiency.

This is consistent with the previous observations of deep learning models trained on limited and heterogeneous ophthalmic datasets that might overfit acquisition-specific cues unless the domain mismatch is clearly addressed [8,9].

Several complementary mechanisms may help explain this comparative robustness. First, the Random Forest decision stage is an ensemble of shallow, decorrelated decision trees, which is inherently less prone to memorising the high-frequency, source-specific texture and illumination patterns that a high-capacity, end-to-end convolutional network may exploit during training. Second, the HOG and LBP descriptors encode local gradient-orientation and micro-texture structure around the optical disc and cup margin, representations that are comparatively invariant to global colour balance and illumination shifts relative to raw pixel intensities learned end-to-end. Third, the mRMR feature-selection stage (Equation (3)) explicitly penalises redundancy among candidate features, discouraging the classifier from relying on a small number of highly correlated, and therefore potentially dataset-specific, feature dimensions. The feature representation ablation in Section 4.2 shows that it is better to use handcrafted HOG and LBP descriptors in the CNN embedding rather than mRMR feature selection and Random Forest classifier as for the performance of Hybrid RF. However, the performance of the CNN + HOG + LBP + mRMR + RF pipeline should still be taken as being the result of the complete CNN + HOG + LBP + mRMR + RF pipeline. The ablation of this sub-sample is done by only feature representation but does not consider the effect of mRMR feature selection or the Random Forest classifier.

The calibration findings add a third and clinically important perspective to the assessment of these models. DAE + TCNN-VGG16 did not outperform Hybrid RF in overall discrimination but had the lowest mean expected calibration error performed across the different sites. Such a difference is clinically relevant since glaucoma screening results are not necessarily binary predictions and can be used to set referral thresholds, triage rules or to support downstream decision making. A model with an acceptable AUC but unstable calibration can still lead to chaotic or unsafe referral behaviour if its probability scale is not reliable across sites. Our findings thus support the argument that discrimination alone is not enough for screening a model and should be applied to all sites, particularly if the deployment is expected to cover a range of institutions or acquisition environments [7,8]. In that sense, the benchmark supports a broader methodological point: probability reliability is not just a secondary performance feature of a model but is a clinical component of the output of a model.

We wish to emphasise, in line with the clinical perspective raised during review, that the models benchmarked here are intended solely as automated screening or triage adjuncts operating on colour fundus photographs, and are not designed or validated to replace a comprehensive ophthalmic examination. Glaucoma diagnosis and management in clinical practice necessarily integrate multiple sources of information beyond a single fundus image, including intraocular pressure measurement, gonioscopy, standard automated perimetry, optical coherence tomography of the retinal nerve fibre layer and ganglion cell complex, family history, and longitudinal structural and functional follow-up. The present benchmark evaluates only the image-based screening component of this broader clinical pathway, under the explicit assumption that a positive or uncertain screening result would prompt referral to a qualified eye-care professional for complete evaluation, rather than an automated diagnostic or treatment decision.

A fourth interesting result is that preprocessing affected performance in a task-dependent rather than uniformly beneficial manner. Raw images had the best mean within-dataset AUC while ROI preprocessing improved the cross-dataset accuracy and Retinex preprocessing improved the cross-dataset MCC. This pattern suggests that preprocessing not only alters visual clarity but also the extent to which a model is dependent on site-specific appearance cues. A preprocessing strategy that focuses on internal discrimination at all costs may reduce transportability if it increases the tonal, geometric, or illumination characteristics of the dataset. In practical terms, this is a reason why image enhancement or ROI restriction are not universally good defaults. Instead, preprocessing decisions should be justified in relation to the intended deployment scenario, particularly if external validity is a central objective.

### Comparison with Prior Results on the Same Datasets

It is instructive to compare our within-dataset findings to previously reported results on the same source datasets, while bearing in mind that direct numerical comparison is complicated by differences in experimental protocol. Mahum et al. [12] reported approximately 99% accuracy on DRISHTI-GS and RIM-ONE using a related hybrid CNN + HOG + LBP + SURF + Random Forest design, and Alghamdi et al. [10] reported 93.8% accuracy and 95% AUC for their SSCNN-DAE model on RIM-ONE. Our corresponding best within-dataset configurations achieve somewhat more conservative values (Table 5 and Table 7). We attribute this difference primarily to methodological factors: our protocol enforces a held-out test partition combined with validation-based, MCC-optimised threshold selection rather than a fixed 0.5 threshold, requires performance to be averaged over five independent random seeds rather than a single favourable split, and applies an identical stochastic augmentation and balanced-sampling policy uniformly across all compared models (Table 4). This comparison illustrates a broader point raised in the literature review: reported accuracy figures across glaucoma-screening studies are not directly comparable unless data partitioning, threshold selection, and evaluation protocol are explicitly matched [1,2,3,16].

Optics and ophthalmology perspective, the domain shift is not surprising. Fundus photographs are affected by camera optics, focus, flash illumination, magnification, vignetting, optic-disc centering, retinal pigmentation, media clarity, and operator acquisition workflow. These factors will influence the observed cup-disc shape, neuroretinal rim contrast, vessel visibility, and peripapillary texture presented to the classifier and will affect handcrafted descriptors and deep representations. This explains why optic disc photography, which can be selected for screening because of its accessibility and low cost, is less objective than OCT-based structural assessment [5] (and thus it should be considered as a test of image-based screening for multimodal glaucoma diagnosis when acquisition variability is not even the minimum prerequisite).

The content of the benchmark datasets should also be considered. Reviews of public glaucoma datasets have pointed out limitations that are relevant to us here; variable diagnostic criteria, incomplete reporting of ground-truth derivation, prevalence distributions that might differ from the real screening context, limited predefined partitions, sparse multimodal clinical metadata, and insufficient representation of acquisition diversity across devices and populations [9]. These limitations do not prohibit the current benchmark but they limit the inference that can be made from it. In particular, many public fundus datasets represent glaucoma as a binary label without a clinical basis for diagnosis, while the usual assessment of glaucoma involves optic disc appearance with OCT, visual field, intraocular pressure, and progression over time. Therefore, this study should be seen as a benchmark of fundus-photo-based screening performance under public-dataset limitations and not as a substitute for multimodal diagnostic assessment.

At the same time, the study has some methodological strengths. It goes beyond single-dataset reporting and uses four public datasets, repeated random seeds, preprocessing ablation, calibration, threshold stability assessment, computational profiling, and non-parametric statistical analysis. These features make the benchmark more informative than studies that report only one or two discrimination metrics from a single split. Statistical analyses of the data also support these results by showing that the main differences between the rankings were systematic and not coincidental. However, there are still a number of limitations in our findings. The study is retrospective, based on public datasets with heterogeneous and sometimes incomplete label provenance, absent any external validation and does not assess formal domain adaptation or multimodal fusion. Furthermore, although the threshold stability was investigated, there was no clinically expected operating point for all datasets, so the threshold results should be viewed as portability and not as a final deployment strategy.

All taken together, these results support one of the main translational findings: glaucoma-screening models should not only be judged on internal accuracy or AUC alone. More clinically relevant criteria include maintaining sensitivity–specificity balance after transfer, calibration reliability, the ability to scale the operating threshold, experiment stability, and the cost of computing. Hybrid RF seems to be the best overall choice when external robustness and efficiency are taken into account, while DAE + TCNN-VGG16 still makes sense when good probability estimation is a priority. Future work should therefore be focused on multi-centred prospective validation, calibration-aware deployment, multimodal label optimisation and domain-aware modelling to keep the diagnosis reliable in various imaging environments.

## 6. Conclusions

We have presented a controlled four-dataset benchmark for fundus-image glaucoma screening using RIM-ONE, DRISHTI-GS, HYGD, and ORIGA, and evaluated the study within a classification and data translation within and across the dataset. In the study, the results were very consistent: great internal performance does not translate to external robustness. Models that achieved good results when training and testing were made from the same distribution and significantly deteriorated when compared across a variety of datasets, highlighting that domain shift is still an issue for clinical-based glaucoma screening AI.

The complete Hybrid RF pipeline is the most discriminating, transportable, and computationally efficient and comparable among the configurations evaluated. It was the best matched-source performance and had the strongest absolute discrimination under cross-dataset transfer and the best overall performance. This can be viewed as a result of the entire CNN + HOG + LBP + mRMR + RF pipeline but not as proof of any single component solely responsible for the observed advantage. In contrast, DAE + TCNN-VGG16 was especially high-quality in calibration data transfer and yielded more reliable probability estimates even though its discrimination was not as good as all of them combined. This is important because screening systems are used not only for classification of images but also to make referrals and triage decisions based on trustworthy probability estimates.

At the same time, the preprocessing also influenced both internal and external performance differently. Raw images were most favorable for within-dataset AUC, ROI preprocessing improved cross-dataset accuracy, and Retinex preprocessing had the best cross-dataset MCC, which means no preprocessing strategy is optimal. Threshold analysis also showed that different operating points were available across datasets, meaning that a threshold tuned to one model cannot be assumed to hold in another clinical environment.

From the translational perspective, the key point is that the current models should not be placed only on internal accuracy or AUC. More important criteria are the preservation of sensitivity–specificity balance after transfer, calibration reliability, threshold portability, experimental stability, and computational practicality. Most importantly, the very small cross-dataset results suggest that none of the models that we have tested are ready for zero-shot deployment in heterogeneous external cohorts without any recalibration, adaptation, or site-specific validation.

We reiterate that, irrespective of future improvements in cross-dataset robustness, such models are intended to support—rather than replace—the clinical judgement of eye-care professionals, who alone can integrate fundus appearance with intraocular pressure, visual field testing, optical coherence tomography, and longitudinal patient history into a complete diagnostic and management decision. Several limitations should be noted: our benchmark is retrospective and is based on public datasets with different label origins and acquisition conditions. We are not considering multimodal structural or functional measurements such as OCT or visual field testing. Future work should concentrate on multicenter prospective validation, calibration-aware deployment, multimodal label refinement, and domain adaptation to guarantee diagnostic reliability in heterogeneous imaging environments.

## Figures and Tables

**Table 1:** Comparison of representative glaucoma-screening studies with respect to deployment-oriented evaluation dimensions. “Yes” indicates that the dimension was explicitly evaluated; “Limited” indicates partial or non-systematic reporting.

Study	Datasets/Modality	Main Approach	Cross-Dataset	Calibration	Threshold Stability	Seeds	Cost
Mahum et al. [12]	DRISHTI-GS, RIM-ONE	Hybrid CNN + handcrafted	No	No	No	No	No
Alghamdi et al. [10]	RIM-ONE, RIGA	Transfer/semi-supervised CNN	No	No	No	No	No
Song et al. [15]	Fundus dataset	Retinex + compact CNN + DoE	No	No	No	Partial	Limited
Govindan et al. [14]	ORIGA, STARE, REFUGE	Transfer learning	Limited	No	No	No	No
Sidhu et al. [18]	Clinical fundus photographs	ResNet50V2	No	No	No	No	Limited
Madadi et al. [8]	OHTS, ACRIMA, RIM-ONE	Domain adaptation	Yes	No	No	No	No
Lenka et al. [19]	Fundus images	Graph/multitask learning	Limited	No	No	No	No
Okuwobi et al. [22]	REFUGE and additional datasets	Deep learning framework	Limited	No	No	No	No
Noh et al. [23]	Fundus images	Transformer/segmentation	Limited	No	No	No	Limited
Bouris et al. [24]	Optic-disc photographs	Vision transformer	Limited	No	No	No	No
Das and Reddy [25]	Retinal fundus images	Attention-guided hybrid features	No	No	No	No	No
Chuter et al. [26]	Fundus + OCT	Multimodal foundation AI	Limited	Limited	No	No	Limited
Majumdar and Mitra [27]	Ophthalmic imaging	Explainable/generative AI review	N/A	Discussed	Discussed	N/A	N/A
El-Gawad et al. [28]	Glaucoma imaging studies	Explainable AI review	N/A	Discussed	Discussed	N/A	N/A
Present study	RIM-ONE, DRISHTI-GS, HYGD, ORIGA	Six model implementations	Yes, 12 transfers	Yes, Brier/ECE	Yes	Yes, 5	Yes

**Table 2:** Summary of the overall experimental design, provided to orient the reader before the detailed description of datasets, preprocessing, and model families that follows.

Dimension	Levels
Datasets (4)	RIM-ONE (485 images), DRISHTI-GS (101 images), HYGD (747 images), ORIGA (650 images)
Model families (3)/models (6)	Hybrid deep–handcrafted RF; VGG16 transfer/semi-supervised family (TCNN-VGG16, SSCNN-Pseudo, DAE + TCNN-VGG16, SSCNN-DAE); Compact Retinex-enhanced CNN
Preprocessing configurations (4)	Raw, ROI, Retinex, ROI + Retinex
Random seeds (5)	0, 1, 2, 3, 4
Evaluation tasks (16)	4 within-dataset + 12 ordered cross-dataset
Metrics	Accuracy, AUC, Balanced Accuracy, MCC, Sensitivity, Specificity, Precision, F1, Brier score, ECE
Statistical testing	Friedman omnibus test + Holm–Bonferroni- corrected Wilcoxon signed-rank tests

**Table 3:** Dataset summary and target split sizes under the 70:10:20 stratified protocol. Exact seed-specific class counts may vary marginally due to integer rounding.

Dataset	Normal	Glaucoma	Total	Train	Val	Test
RIM-ONE	313	172	485	339	49	97
DRISHTI-GS	31	70	101	71	9	21
HYGD *	199	548	747	522	75	150
ORIGA	482	168	650	454	65	131

* HYGD labels GON- and GON+ were mapped to normal and glaucoma, respectively, before partitioning.

**Table 4:** Training configuration and hyperparameters shared across the benchmark.

Parameter	Value	Applies to
Random seeds	{0,1,2,3,4}	All frameworks
Image input size	224×224 px	All frameworks
Labelled ratio (SSL)	0.6	SSCNN, SSCNN-DAE
Pseudo-label threshold	0.9	SSCNN-Pseudo, SSCNN-DAE
Loss function	Weighted BCE	Deep learning frameworks
Class weights	Inverse class frequency	Deep learning frameworks
Balanced sampler	Enabled	Deep learning frameworks
Epochs (main)	20	Deep learning frameworks
Epochs (DAE pre-train)	8	DAE + TCNN, SSCNN-DAE
Threshold metric	MCC (validation set)	All frameworks
TTA views	*K* stochastic transforms	All frameworks
RF trees	Benchmark default	Hybrid RF
DOE search	Taguchi/factorial	Compact CNN

**Table 5:** Headline benchmark findings for the leading models under within-dataset and cross-dataset evaluation. Within-dataset values summarize the strongest overall matched-source performance reported in the manuscript, whereas cross-dataset values summarize the leading transport results and calibration findings under external transfer.

Evaluation	Model	Accuracy	AUC	MCC	Balanced Acc.	Brier	ECE
Within-dataset	Hybrid RF	0.8697	0.8972	0.6983	0.8428	0.1202	0.1621
Cross-dataset	Hybrid RF	0.5685	0.5798	0.0871	0.5429	–	–
Cross-dataset	DAE + TCNN-VGG16	0.5424	–	–	–	–	0.2504

**Table 6:** Best available within-dataset configuration for each model. Each row corresponds to the highest-performing within-dataset configuration identified for that model.

Model	Dataset	Preprocess	Accuracy	AUC	MCC	Balanced Acc.	Brier	ECE
CompactCNN	HYGD	raw	0.9101	0.9693	0.8015	0.9259	0.3012	0.4360
DAE + TCNN-VGG16	RIM-ONE	roi_retinex	0.7474	0.8130	0.4725	0.7358	0.2414	0.2467
Hybrid RF	HYGD	raw	0.9503	0.9853	0.8758	0.9407	0.0460	0.0915
SSCNN-DAE	HYGD	retinex	0.8711	0.9370	0.6926	0.8597	0.3469	0.4537
SSCNN-Pseudo	HYGD	raw	0.9570	0.9833	0.8935	0.9548	0.0669	0.0930
TCNN-VGG16	HYGD	raw	0.9517	0.9863	0.8806	0.9480	0.0778	0.1222

**Table 7:** Best within-dataset result per dataset, ranked by AUC. This table provides a compact dataset-level summary of the strongest matched-source performer for each benchmark cohort.

Dataset	Winning Model	Preprocess	Accuracy	AUC	MCC	Balanced Acc.
RIM-ONE	Hybrid RF	retinex	0.9423	0.9887	0.8763	0.9344
DRISHTI-GS	Hybrid RF	raw	0.8500	0.8274	0.6469	0.7881
HYGD	TCNN-VGG16	raw	0.9517	0.9863	0.8806	0.9480
ORIGA	Hybrid RF	raw	0.7333	0.7862	0.3863	0.7028

**Table 8:** Feature-representation ablation of the hybrid deep–handcrafted framework. Values represent mean accuracy and AUC across all four datasets and five random seeds under the raw preprocessing configuration. The mRMR feature-selection stage and Random Forest classifier were held fixed across all rows. The full CNN + HOG + LBP configuration corresponds to the Hybrid RF model reported elsewhere in this study.

Feature Configuration	Within Acc.	Within AUC	Cross Acc.	Cross AUC
CNN only	0.8310	0.8615	0.5312	0.5486
CNN + HOG	0.8508	0.8819	0.5504	0.5668
CNN + LBP	0.8449	0.8756	0.5437	0.5607
CNN + HOG + LBP (full)	0.8697	0.8972	0.5685	0.5798

**Table 9:** Best cross-dataset transfer result for each ordered source→target pair, ranked by AUC. This table makes the domain-shift difficulty of each transfer direction numerically explicit.

Train	Test	Model	Preprocess	Accuracy	AUC	MCC	Balanced Acc.
RIM-ONE	ORIGA	TCNN-VGG16	roi	0.3231	0.7659	0.1514	0.5448
DRISHTI-GS	RIM-ONE	Hybrid RF	raw	0.6495	0.6674	0.2090	0.5923
DRISHTI-GS	HYGD	CompactCNN	retinex	0.6255	0.6467	0.2331	0.6301
ORIGA	RIM-ONE	SSCNN-Pseudo	retinex	0.5619	0.6163	0.2012	0.5905
HYGD	DRISHTI-GS	SSCNN-Pseudo	raw	0.6300	0.5952	0.0694	0.5262
RIM-ONE	DRISHTI-GS	Hybrid RF	raw	0.4000	0.5738	−0.0236	0.4952
HYGD	RIM-ONE	DAE + TCNN-VGG16	raw	0.4835	0.5447	−0.0039	0.4979
RIM-ONE	HYGD	SSCNN-DAE	roi_retinex	0.7356	0.5425	0.0595	0.5091
HYGD	ORIGA	DAE + TCNN-VGG16	raw	0.5026	0.5421	0.1477	0.5738
DRISHTI-GS	ORIGA	CompactCNN	raw	0.6308	0.5179	0.0137	0.5093
ORIGA	DRISHTI-GS	SSCNN-Pseudo	raw	0.7000	0.4810	0.0000	0.5000
ORIGA	HYGD	TCNN-VGG16	roi_retinex	0.7235	0.3806	−0.0171	0.4961

**Table 10:** Generalisation gap by model. Larger Δ values indicate greater loss of performance when moving from within-dataset evaluation to cross-dataset transfer.

Model	Within AUC	Cross AUC	ΔAUC	Within Bal. Acc.	Cross Bal. Acc.	ΔBal. Acc.
DAE + TCNN-VGG16	0.6758	0.4907	0.1851	0.6301	0.4998	0.1303
SSCNN-DAE	0.6852	0.4960	0.1892	0.6211	0.4999	0.1213
CompactCNN	0.7520	0.5038	0.2483	0.7019	0.5002	0.2017
SSCNN-Pseudo	0.7795	0.4921	0.2874	0.7336	0.4946	0.2390
TCNN-VGG16	0.7959	0.4683	0.3276	0.7426	0.4984	0.2441
Hybrid RF	0.8900	0.5589	0.3310	0.8292	0.5262	0.3030

**Table 11:** Model-level calibration and threshold summary. Lower Brier and ECE values indicate better-calibrated probability estimates.

Evaluation	Model	Mean Threshold	Mean Brier	Mean ECE
Within-dataset	Hybrid RF	0.49	0.12	0.15
Within-dataset	TCNN-VGG16	0.44	0.24	0.27
Within-dataset	SSCNN-Pseudo	0.45	0.21	0.24
Within-dataset	DAE + TCNN-VGG16	0.48	0.35	0.40
Within-dataset	SSCNN-DAE	0.46	0.33	0.38
Within-dataset	CompactCNN	0.41	0.26	0.30
Cross-dataset	Hybrid RF	0.44	0.28	0.25
Cross-dataset	TCNN-VGG16	0.48	0.40	0.41
Cross-dataset	SSCNN-Pseudo	0.50	0.41	0.42
Cross-dataset	DAE + TCNN-VGG16	0.51	0.30	0.25
Cross-dataset	SSCNN-DAE	0.49	0.31	0.29
Cross-dataset	CompactCNN	0.48	0.36	0.36

**Table 12:** Threshold portability across within-dataset and cross-dataset evaluation. Lower |Δτ| indicates greater stability of the selected operating threshold across domains.

Model	Within τ	Cross τ	|Δτ|	Within ECE	Cross ECE
DAE + TCNN-VGG16	0.4813	0.4771	0.0042	0.3870	0.2729
TCNN-VGG16	0.4631	0.4573	0.0058	0.2591	0.4178
Hybrid RF	0.4919	0.4850	0.0069	0.1608	0.2709
CompactCNN	0.4775	0.4644	0.0131	0.3205	0.3585
SSCNN-DAE	0.4631	0.4817	0.0185	0.3771	0.2949
SSCNN-Pseudo	0.4625	0.4433	0.0192	0.2467	0.4517

**Table 13:** Representative computational-efficiency summary for the best within-dataset configuration of each model. Parameter count is reported in millions (M).

Model	Params (M)	Train/Epoch (s)	Total Train (min)	Inference (ms)
CompactCNN	0.3896	8.69	3.23	12.84
DAE + TCNN-VGG16	0.5613	8.42	4.19	12.75
Hybrid RF	2.0076	29.22	0.49	0.16
SSCNN-DAE	0.5613	30.98	29.00	68.04
SSCNN-Pseudo	134.2731	11.65	8.49	12.90
TCNN-VGG16	134.2731	11.99	4.35	12.85

**Table 14:** Statistical significance summary. Panel A reports omnibus Friedman tests; Panel B reports representative significant post-hoc Wilcoxon comparisons with Holm correction.

**Panel A. Friedman Omnibus Tests**			
**Evaluation**	**Metric**	**Friedman Statistic**	***p*** **-Value**
Cross-dataset	Accuracy	79.7257	$9.58\times 10^{-16}$
Within-dataset	Accuracy	162.9512	$2.33\times 10^{-33}$
Cross-dataset	AUC	49.3313	$1.90\times 10^{-9}$
Within-dataset	AUC	152.8190	$3.35\times 10^{-31}$
**Panel B. Representative Wilcoxon Comparisons**			
**Metric**	**Comparison**	**Holm-Adjusted *p***	**Mean Δ**
Cross Acc.	Hybrid RF vs. SSCNN-Pseudo	$5.68\times 10^{-14}$	+0.1092
Cross Acc.	Hybrid RF vs. TCNN-VGG16	$2.07\times 10^{-7}$	+0.0774
Cross AUC	Hybrid RF vs. TCNN-VGG16	$2.59\times 10^{-10}$	+0.0906
Cross AUC	Hybrid RF vs. SSCNN-DAE	$1.19\times 10^{-6}$	+0.0629
Within Acc.	Hybrid RF vs. SSCNN-DAE	$8.28\times 10^{-13}$	+0.2132
Within AUC	Hybrid RF vs. DAE + TCNN	$7.01\times 10^{-12}$	+0.2142

## Data Availability

The data presented in this study are available in the article. Further inquiries may be directed to the corresponding author.
